# Geochemical Characteristics and Risk Assessment of PTEs in the Supergene Environment of the Former Zoige Uranium Mine

**DOI:** 10.3390/toxics13070561

**Published:** 2025-06-30

**Authors:** Na Zhang, Zeming Shi, Chengjie Zou, Yinghai Zhu, Yun Hou

**Affiliations:** 1 College of Earth Sciences, Chengdu University of Technology, Chengdu 610059, China; zhangnana1115@stu.cdut.edu.cn (N.Z.); zou@stu.cdut.edu.cn (C.Z.); zhuyinghai@stu.cdut.edu.cn (Y.Z.); hou_yun@163.com (Y.H.); 2 Sichuan Province Key Laboratory of Nuclear Techniques in Geosciences, Chengdu 610059, China

**Keywords:** Zoige uranium mine, PTEs, sequential extraction, source apportionment, ecological health risk, sediment, soil

## Abstract

Carbonaceous–siliceous–argillaceous rock-type uranium deposits, a major uranium resource in China, pose significant environmental risks due to heavy metal contamination. Geochemical investigations in the former Zoige uranium mine revealed elevated As, Cd, Cr, Cu, Ni, U, and Zn concentrations in soils and sediments, particularly at river confluences and downstream regions, attributed to leachate migration from ore bodies and tailings ponds. Surface samples exhibited high Cd bioavailability. The integrated BCR and mineral analysis reveals that Acid-soluble and reducible fractions of Ni, Cu, Zn, As, and Pb are governed by carbonate dissolution and Fe-Mn oxide dynamics via silicate weathering, while residual and oxidizable fractions show weak mineral-phase dependencies. Positive Matrix Factorization identified natural lithogenic, anthropogenic–natural composite, mining-related sources. Pollution assessments using geo-accumulation index and contamination factor demonstrated severe contamination disparities: soils showed extreme Cd pollution, moderate U, As, Zn contamination, and no Cr, Pb pollution (overall moderate risk); sediments exhibited extreme Cd pollution, moderate Ni, Zn, U levels, and negligible Cr, Pb impacts (overall extreme risk). USEPA health risk models indicated notable non-carcinogenic (higher in adults) and carcinogenic risks (higher in children) for both age groups. Ecological risk assessments categorized As, Cr, Cu, Ni, Pb, and Zn as low risk, contrasting with Cd (extremely high risk) and sediment-bound U (high risk). These findings underscore mining legacy as a critical environmental stressor and highlight the necessity for multi-source pollution mitigation strategies.

## 1. Introduction

The types of uranium deposits in China can be primarily categorized into four major groups: volcanic-type uranium deposits, sandstone-type uranium deposits, granite-type uranium deposits, and carbonate–siliceous–pelitic type uranium deposits [1]. Different uranium mining technologies induce varying degrees of ecological risks in mining areas. Conventional uranium extraction techniques include open-pit mining, underground mining, and in situ heap leaching. Among these, sandstone-type uranium deposits predominantly employ in situ leaching methods, while hard-rock type uranium deposits mainly utilize heap leaching techniques. In situ leaching involves injecting leaching agents such as oxidizing agents or complexing carrier solvents into uranium-bearing strata. These agents oxidize tetravalent uranium (U(IV)) in ores into soluble hexavalent uranium (U(VI)), or enhance uranium dissolution through complexation with uranyl ions (UO_2_^2+^). In contrast, heap leaching requires preliminary excavation and blasting to extract uranium ore, followed by selective leaching of valuable components through chemical solution irrigation. Notably, in situ leaching carries higher risks of contaminating aquifers and deep subsurface environments, whereas heap leaching demonstrates greater potential for polluting surface water systems, sediments, and topsoil [2].

Uranium-contaminated soils have been documented globally, though contamination characteristics show significant regional variations. In southwestern China’s uranium mining regions, soils exhibit average uranium concentrations of 19.62 mg/kg with pollution load indices exceeding 5, indicating severe heavy metal contamination (including uranium) intensified by mining operations [3]. The Cunha Baixa uranium mine in central Portugal presents soils with exceptionally high ecological risks [4]. At France’s Rophin mining district, historical exploitation of metallic deposits has caused persistent contamination of river systems and surrounding environments with radionuclides and heavy metals, significantly altering microbial community structures [5,6]. Heavy metal contamination represents a global environmental challenge due to these elements’ environmental persistence and inherent toxicity to organisms at specific concentration thresholds [7].

The Zoige uranium mining area underwent exploration and extraction between 1970 and 1990 and has remained abandoned for over three decades. Its mining operations predominantly employed open-pit methods, while heap leaching techniques—known for higher contamination risks to surface/near-surface soils and water bodies—were also utilized. Previous studies have extensively documented radionuclides and heavy metals in groundwater/surface water, soils, and sediments from China’s volcanic-type, granite-type, and sandstone-type uranium deposits [8,9,10,11,12,13]. However, research on heavy metal contamination in soils and sediments from carbonate–siliceous–pelitic rock-type uranium deposits in plateau regions remains scarce. These contaminants, comprising heavy metals and radioactive elements, pose dual threats through chemical toxicity and radiotoxicity, jeopardizing the growth, development, and reproduction of flora and fauna while accumulating through food chains to endanger human health [14]. The Zoige wetland’s ecological significance is magnified by its unique geographical position as a headwater conservation and recharge zone for the Upper Yellow River Basin and its status as an organic-rich plateau wetland, rendering its potential environmental impacts exceptionally profound.

To better evaluate the ecological impacts of heavy metals from the Zoige carbonate–siliceous–pelitic rock-type uranium deposit, it is imperative to investigate the geochemical characteristics and risk assessment of heavy metals in local soils and sediments. This study focuses on the abandoned Zoige uranium mining area. Building on the identification of heavy metal contamination levels and mineralogical features in regional soils and sediments, we employ the Positive Matrix Factorization (PMF) model for source apportionment. Ecological risks are systematically evaluated using the geo-accumulation index (Igeo), pollution load index (PLI), and PERI (RI). Additionally, the US Environmental Protection Agency (USEPA) health risk assessment model is applied to quantify non-carcinogenic and carcinogenic risks posed by soil heavy metals. This integrated approach aims to do the following: (1) Determine the concentrations and contamination extent of heavy metals in surface soils and sediments; (2) Identify the ecological risks associated with historical uranium mining activities; (3) Assess their potential impacts on human health. The findings are expected to provide critical scientific foundations for ecological remediation and environmental management strategies in the Zoige uranium mining region.

## 2. Materials and Methods

### 2.1. Site Description and Sample Sampling

The study area is located in Jiangzha Township, Zoige County, Sichuan Province, China. Situated in the northern part of the county approximately 90 km from the county seat, it has an average elevation of 3044 m above sea level. The region experiences a mean annual temperature of 3 °C. Demographic data indicate a total population of 3010 inhabitants, with 2939 permanent residents. The carbonate–siliceous–pelitic rock-type uranium field in the Zoige region represents a strategically significant uranium mining district in China. Spanning the border area between Zoige County (Aba Tibetan and Qiang Autonomous Prefecture, northwestern Sichuan) and Diebu/Luqu Counties (Gannan Tibetan Autonomous Prefecture, Gansu Province), this metallogenic zone extends approximately 50 km east–west and 6 km north–south. Situated on the northwestern Sichuan Plateau, the mining district hosts multiple large, medium, and small uranium deposits. Notably, its carbonate–siliceous–pelitic rock-type uranium ores not only exhibit high uranium grades, but also contain associated metal elements such as Zn, V, Cu, Cr, Mn, and Ni, offering potential for comprehensive utilization. These distinctive characteristics have attracted considerable attention from geological researchers [15,16,17].

Samples were collected in August 2022 from six sites within the Zoige mining area (102°45′–102°46′ E, 34°12′–34°13′ N): Xiangyang West (XW), Xiangyang East (XE), Zhongchanggou (ZC), Yangchanggou (YC), Qielugou (QL), and Sulimugou (SL).

Zones I–V: Located distally from primary transportation corridors, these areas exhibit sparse residential populations and are predominantly utilized for livestock husbandry.

Zone III: Previously served as the principal operational sector of the Ruo’ergai uranium mine, which has remained abandoned for three decades since cessation of activities.

Zone IV: Designated as the tailing disposal area, where uranium tailings stockpiles persist to this day, demonstrating measurable residual radioactivity.

Zone VI: Encompasses the main channel of the Heihe River. This area sustains a substantial permanent population and experiences significant tourist influx due to the proximal Jiangzha Hot Springs located in its downstream reaches.

**Figure 1:**
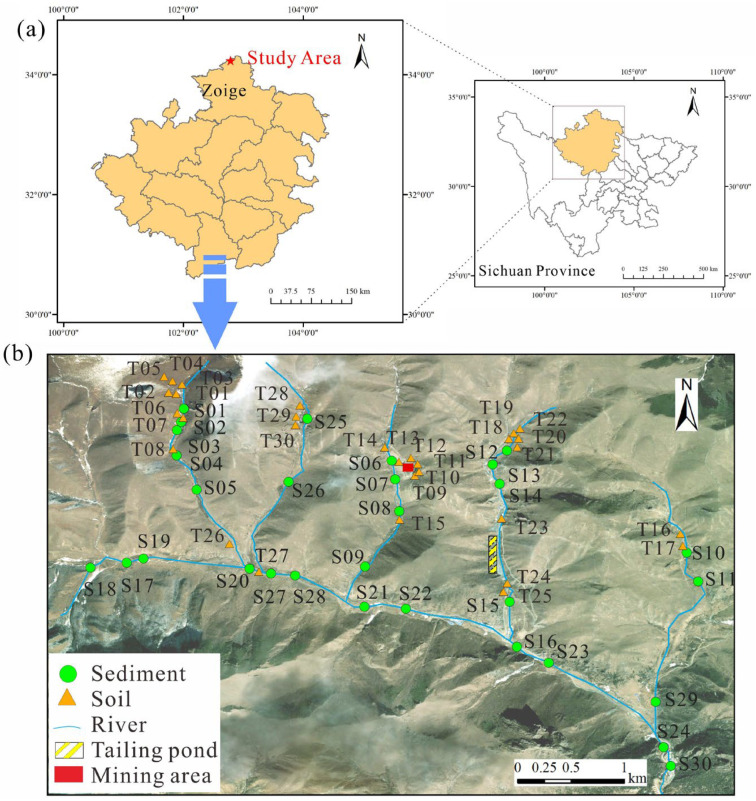
Sampling map of the study area. (**a**) Geographical location map; (**b**) sample collection map.

Situated at elevations of 3300–3700 m above sea level, all sampling locations followed the natural flow direction from upstream to downstream. Initial fieldwork involved GIS-based geolocation mapping, yielding 30 representative soil and fluvial sediment sampling points (Figure 1). Surface soil samples (5–20 cm depth) were collected using a standardized protocol: a 2 m^2^ quadrat was established at each site, with each composite sample comprising two subsamples. After removing coarse gravel (>2 mm) and plant debris, subsamples were homogenized through quartering. All specimens were sealed in polyethylene bags and transported to the laboratory under chilled conditions (4 °C) to preserve geochemical integrity.

### 2.2. Sample Analysis

#### 2.2.1. BCR Sequential Extraction Procedure

The chemical speciation of eight PTEs in soils and sediments was determined using a modified BCR (Community Bureau of Reference) sequential extraction protocol [2,18,19]. All analytical procedures were conducted at the Sichuan Provincial Key Laboratory of Nuclear Technology in Geosciences, with sample measurements performed via inductively coupled plasma mass spectrometry (ICP-MS). Rigorous quality control measures were implemented, including the incorporation of method blanks, duplicate samples, and certified reference materials (CRMs). Calibration curves demonstrated excellent linearity (coefficient of determination R^2^ > 0.999), while recoveries for the soil reference material (GSS-27) ranged between 90% and 110%. Analytical precision was maintained with relative standard deviations (RSDs) ≤ 5%. The experimental protocols were documented in Table 1

#### 2.2.2. Elemental Analysis

The bulk compositional analysis of the samples was conducted in the ALS laboratory group (ALS Minerals–ALS Chemex, Brisbane, Australia) in Guangzhou. Major element analysis was performed by X-ray fluorescence spectrometry (XRF, PANalytical Axios Adv PW4400, Almelo, the Netherlands). After the samples were digested with a mixture of HCl, HNO_3_, HF, and HClO_4_, trace elements were measured using an inductively coupled plasma mass spectrometer (ICP-MS, Agilent 5110, Santa Clara, CA, USA). Quality control was performed using blanks, replicates, and reference materials. For all samples, the analytical results of the reference material were within ±10% of the certified value, and the relative standard deviation (RSD) value of the replicates was within 5%.

#### 2.2.3. pH Determination in Soils and Sediments

Soil pH measurements were conducted in strict compliance with the Potentiometric Method for Determination of Soil pH (Chinese EPA Standard HJ 962-2018). Precisely weigh 10.00 ± 0.05 g air-dried sample (<2 mm) into 50 mL borosilicate beaker. Add 25 mL Type I ultrapure water at soil-to-water ratio 1:2.5 (m:v). Homogenize with PTFE-coated stir rod (15 clockwise + 15 counter-clockwise rotations). Allow suspension to stand at 25 ± 2 °C (ambient conditions) for 30 ± 1 min. At the same time, calibrate the pH meter using the pH standard buffer solution. Measure the pH with the machine after preparatory work is completed.

#### 2.2.4. X-Ray Diffraction

Mineralogical characterization was conducted through X-ray diffraction (XRD) analysis using a BRUKER-D8 ADVANCE diffractometer (Hannover, Germany). Prior to analysis, samples were pulverized to particle sizes < 40 μm using an agate mortar. XRD measurements employed Cu Kα radiation generated at 36 kV and 36 mA, with continuous scanning across a 2θ range of 5–70° to ensure comprehensive phase detection. Mineral identification was achieved by matching characteristic diffraction peaks against the International Centre for Diffraction Data (ICDD) PDF-4+ database. Semi-quantitative phase abundance was determined through Rietveld refinement using the Whole Pattern Fitting (WPF) module in JADE 9.0 software (Materials Data, Inc, Livermore, CA, USA).

#### 2.2.5. Scanning Electron Microscope–Energy Dispersive X-Ray Spectroscopy

Initial mineralogical characterization was performed using polarizing microscopy to examine thin sections, enabling identification of mineral assemblages and textural relationships. Target areas for subsequent microanalysis were identified and marked with a solvent-resistant pen. Selected specimens were sputter-coated with 10 nm Au/Pd alloy using a high-vacuum coater (Q150T ES, Quorum Technologies) to enhance surface conductivity and prevent charging effects. Surface morphology observation and energy spectrum analysis of samples were Conducted by FEI Nova Nano SEM 450 field-emission scanning electron microscope equipped with an Octane Elite EDS system (EDAX).

### 2.3. Risk Assessment

#### 2.3.1. Geo-Accumulation Index

The geo-accumulation index (I_geo_), originally proposed by Müller [20] to quantitatively assess heavy metal contamination in sediments, has become a widely adopted method for evaluating pollutant enrichment in soils, sediments, atmospheric deposition, and other environmental media. Table 2 is Classification of geo-accumulation index (Igeo) in relation to soil quality.

#### 2.3.2. Contamination Factor

The Contamination factor (CF), initially developed by Tomlinson [21] for assessing heavy metal contamination in estuarine sediments, has evolved into a robust quantitative method for visualizing spatial trends of multi-element pollution in environmental matrices. The PLI is calculated as the following:

The pollution factor of heavy metal *i* at Point *j* is

(1) $${CF}_{\mathrm{ij}}=C_{ij}/B_{i}$$

The pollution load index of point *j* is

(2) $${PLI}_{j}=\sqrt[{m}]{{CF}_{ij\;}\;\times \;{CF}_{2j\;}\times \;{CF}_{3j\;}\times \;\cdots \;\times \;{CF}_{\mathrm{mj}}}$$

The pollution load index of a certain region is

(3) $${PLI}_{Zone}=\sqrt[{n}]{{PLI}_{1}\;\times {\;PLI}_{2}\;\times {\;PLI}_{3}\;\times \;\cdots \;\times \;{PLI}_{n}}$$

where *C_ij_* is the concentration of concerned heavy metal *i* at Point *j*; *B_i_* is the corresponding geochemical background value; *m* is the number of analyzed PTEs and *n* is the number of sampling points. The classification was showed in Table 3.

#### 2.3.3. Potential Ecological Risk Index

The Potential Ecological Risk Index (PERI) methodology, developed by Hakanson [22], addresses the need for comprehensive assessment of combined heavy metal toxicity by integrating multiple critical factors. The RI is calculated as

(4) $$RI=\sum_{i=1}^{n}E_{r}^{i}=\sum_{i=1}^{n}\left(T_{r}^{i}\times C_{r}^{i}\right)=\sum_{i=1}^{n}\left(T_{i}\times \frac{C_{s}^{i}}{C_{n}^{i}}\right)$$

where *RI* is the Potential Ecological Risk Index; $E_{r}^{i}$ is the ecological risk index factor of heavy metal element *i*; $T_{r}^{i}$ is the toxic response factor of heavy metal *i*; $C_{r}^{i}$ is the pollution index of heavy metal *i*; $C_{s}^{i}$ is the measured value for heavy metal *i* (mg/kg); and $C_{n}^{i}$ is the local background value of heavy metal *i* (mg/kg). The toxic response factors for PTEs As, Cd, Cr, Cu, Ni, Pb, Zn and U are 10, 30, 2, 5, 5, 5, 1 and 20, respectively. Evaluation criteria for potential ecological risk indices are contained in Table 4.

### 2.4. The Positive Matrix Factorization

The Positive Matrix Factorization (PMF) model is a receptor-oriented analysis model for pollution source apportionment. Distinguished from conventional receptor models, PMF employs a weighted least-squares algorithm with iterative optimization [23]. The original dataset is decomposed by the PMF model into three matrices: factor assessment (gik), factor profile (fkj) and residual matrix (eij). Uncertainty estimation is introduced for each data, and factors are extracted to identify pollution source classes. PMF has become a gold-standard tool for multi-media source apportionment, successfully applied to atmospheric particulates, aquatic systems, sediments, and contaminated soils. Its capacity to resolve collinear sources and quantify source-specific contributions makes it particularly valuable for complex mining-impacted environments.

### 2.5. Human Health Risk Assessment Models

The United States Environmental Protection Agency (USEPA) developed the Human Health Risk Assessment (HHRA) model to systematically evaluate health risks posed by environmental contaminants [24]. This probabilistic framework quantifies potential risks through two critical dimensions: the pollution status of the environment, including the content, migration characteristics, and toxicity of PTEs; and the exposure pathways of soil PTEs, which are oral ingestion (ing), dermal contact (der), and inhalation (inh) in Table 5. The model incorporates demographic-specific parameters to address physiological and behavioral differences between adults and children. Full derivation of calculation equations is provided in the Supplementary Materials.

The Hazard Quotient (HQ) represents ther non-carcinogenic risk from individual PTEs via specific exposure pathways (ingestion, inhalation, and dermal). The Hazard Index (HI) is the summation of HQs across all analyzed metals, representing total non-carcinogenic risk. When *HI* <  1, the risk to the exposed population is considered low and can be ignored; when *HI* >  1, there is a non-carcinogenic health risk. The total carcinogenic risk index of a single heavy metal element under three pathways was expressed as CR, and the total carcinogenic risk index of multiple PTEs was expressed as TCR. When CR/TCR < 1 × 10^−6^, there is negligible carcinogenic risk; when 1 × 10^−6^ ≤ *CR*/*TCR* < 1 × 10^−4^, there is potential carcinogenic concern; when *CR*/*TCR* ≥ 1 × 10^−4^, there is significant carcinogenic risk [29].

## 3. Results and Discussion

### 3.1. Geochemistry Characteristics of PTEs in Soils and Sediments

In Table 6, the surface soil in the study area is generally weakly alkaline, with a pH range of 6.18–7.90 and an average value of 7.03. Except for the Cr and Pb elements, the average content of As, Cd, Cu, Ni, Zn, and U elements exceeds the soil background value in Sichuan Province [30], which is 2.46, 18.00, 1.42, 1.52, 2.33, and 4.53 times higher than the background value. The coefficient of variation of Cr and Pb is less than 0.20, indicating weak variation, which suggests that Cr and Pb have a relatively uniform spatial distribution in the soil of the Zoige uranium mining area. The coefficients of variation for As, Cd, Cu, and Ni are greater than 0.5, indicating strong variability. The coefficients of variation for Zn and U are greater than 1, indicating abnormal strong variability. This suggests that there is a local aggregation phenomenon of As, Cd, Cu, Ni, Zn, and U content in the surface soil of the study area, and their spatial distribution is uneven. In addition to being controlled by geological background, they may also be influenced by human activities. To ensure the quality and safety of agricultural products and normal growth of crops, risk screening values (RSVs) of soil were developed by the Ministry of Environmental Protection of China, and the descriptive statistics are listed in Table 6.

The descriptive statistical results of heavy metal element content in the sediment of the Zoige uranium mining area are shown in Table 7. Among them, the average contents of As, Cd, Cu, Ni, U, and Zn elements exceeded the soil background values in Sichuan Province, which were 3.41, 65.63, 1.82, 4.40, 5.53, and 7.47 times the background values, respectively. Compared with the screening value for soil pollution risk in agricultural land [31], the average content of As, Cd, Ni, and Zn elements in water sediment is higher, while the average content of other elements is lower than the background value. The exceedance rate of As is 63.33%, Cd is 100%, Ni is 36.67%, and Zn is 76.67%. According to Table 7, the coefficient of variation of Cr and Pb in the sediment of the Zoige uranium mining area is less than 0.20, indicating weak variation. This suggests that the spatial distribution of Cr and Pb in the sediment of the Zoige uranium mining area is relatively uniform. The coefficients of variation for As, Cd, Cu, Ni, and Zn are all greater than 0.5, indicating strong variability. The coefficient of variation of Zn and U is greater than 1, indicating an abnormal strong variation and indicating local aggregation of As, Cd, Cu, Ni, Zn, and U contents in the study area. The spatial distribution of water series sediments in the Zoige uranium mining area is uneven, which may be influenced not only by the geological background, but also by human activities.

The concentrations of eight PTEs in the soil are higher than those in the Bayanwula uranium mining area and a uranium mining area in East China in Table 7. The average uranium content in the Nuheting uranium mining area, a uranium mining area in northern Guangdong, and the Xiazhuang uranium mining area is higher than that in the study area, being 4.82 times, 2.42 times, and 2.51 times that of the study area, respectively. The arsenic content in a uranium mining area in northern Guangdong and a uranium tailings area in northwest China is significantly higher, being 36.57 times and 5.25 times that of the study area, respectively. The uranium content in a uranium tailings area in South China is similar to that in the Zoige uranium mining area. Among the eight PTEs in the study area, the highest concentrations are observed for As, Cd, Zn, and U, indicating significant impacts of the Zoige uranium mining area on these four elements. Based on the average heavy metal concentrations in the soil of this study, comparisons were made with the soil background values of Sichuan Province and China, and K_1_ (the ratio of heavy metal concentrations in the surface soil of the study area to the Sichuan soil background values), and K_2_ (the ratio of heavy metal concentrations in the surface soil of the study area to the Chinese soil background values) were calculated. The results show that, except for Cr and Pb, the remaining PTEs exhibit varying degrees of accumulation, with Cd and U showing the highest levels of accumulation.

The concentrations of eight PTEs in the sediments are higher than those in a uranium mining area in East China, a uranium mining area in northern Guangdong, and a uranium mining area in Jiangxi. The concentrations of Cr, Ni, and Pb in the Beishanhe uranium tailings area are higher than those in the Zoige uranium mining area, being 1.56 times, 2.09 times, and 2.16 times those of the study area, respectively. Based on the soil background values of Sichuan Province and China, K_1_ (the ratio of the average heavy metal concentrations in the sediments of the study area to the Sichuan soil background values), K_2_ (the ratio of the average heavy metal concentrations in the sediments of the study area to the Chinese soil background values), and K_3_ (the ratio of the average heavy metal concentrations in the sediments of the study area to the national sediment background values) were calculated. The results show that, except for Cr and Pb, the remaining PTEs exhibit varying degrees of accumulation

**Figure 2:**
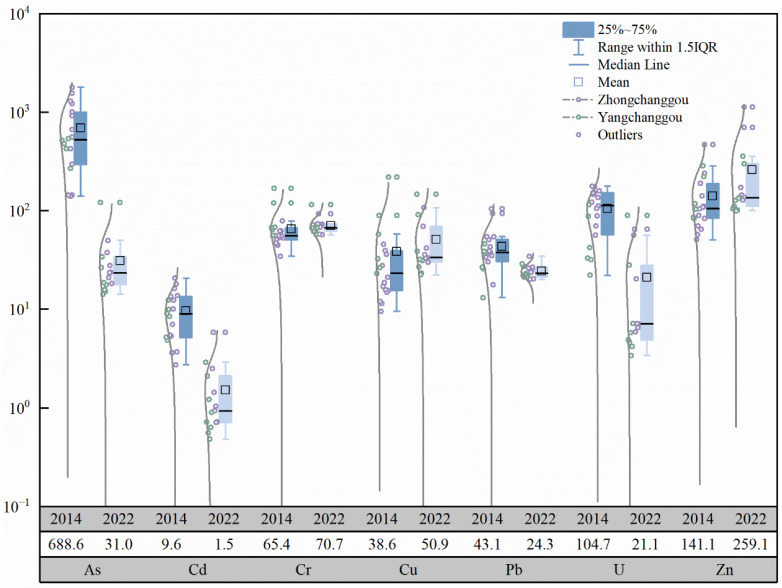
Comparison of soil heavy metal contents in Zoige uranium mining area in 2014 and 2022. The shade represent years and elements respectively, while the white column indicates mean values. The dark blue bars represent elemental concentrations in 2014, while light blue denotes those from 2022.

Based on the data from this study and previous research [43], boxplots of heavy metal concentrations in the Zone Ⅲ and Zone Ⅳ of the Zoige uranium mining area in 2014 and 2022 were plotted, as shown in Figure 2. Compared with previous studies, the concentrations of As, Cd, Pb, and U in the surface soil in 2022 decreased compared to 2014, with As and U showing the most significant reductions, while Cr, Cu, and Pb showed little difference. Compared to 2014, the reductions in As, Cd, Pb, and U concentrations in the surface soil of Zone Ⅲ were greater than those in Zone Ⅳ, while the increases in Cu and Zn concentrations in the Zone Ⅲ were greater than those in Zone Ⅳ, and the changes in Cr and Pb were minimal. In 2022, the abnormally high values of As, Cd, Cr, Cu, Ni, Pb, Zn, and U in the surface soil of the study area did not appear at the sampling points T09, T10, T11, T12, and T13, around the mining area. Based on field investigations, it is speculated that the former open-pit mining area may have been sealed with cement engineering measures to prevent the migration of uranium and other PTEs, while biological reclamation measures may have also contributed to the remediation of the soil around the mining area.

### 3.2. Sequential Extraction of PTEs

**Figure 3:**
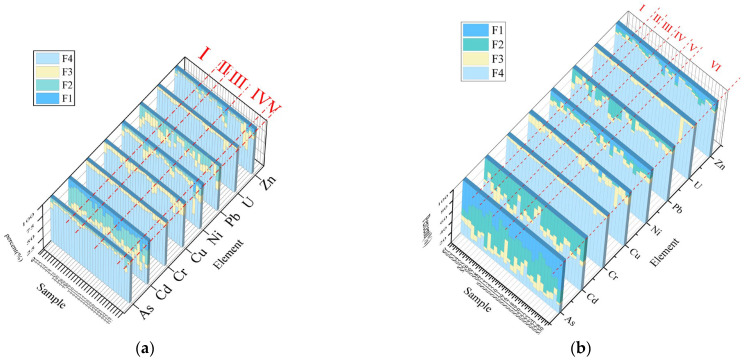
Distribution characteristics of heavy metal forms. (**a**) Soil; (**b**) sediment. Note: weak acid-extractable fraction (F1), reducible fraction (F2), oxidizable fraction (F3), and residual fraction (F4); n = 30.

As shown in Figure 3, significant variations exist in the chemical speciation of PTEs across sampling sites. Cadmium predominantly occurs in the high F1 fraction (carbonate-bound and water-soluble forms), indicating elevated bioavailability and ecological risks. The elevated F2 fractions (Fe-Mn oxide-bound) of Cd and Pb in accumulation Zone IV are attributed to local tailings deposits containing abundant Fe-Mn elements, as Pb and Cd exhibit preferential binding with Fe-Mn oxides compared to other metals (e.g., Cu, Zn) [44]. Consequently, the dominance of F2 fractions for Cd, Pb in Zones I–V and Zn in Zone III suggests Fe-Mn oxides predominantly control these metals beyond residual forms.

Regarding organic-bound fractions (F3), Cd, Pb, Cu, U, and Zn demonstrate substantial proportions across all zones, reflecting strong organic matter control. Overall, As, Cr, Cu, Ni, Pb, U, and Zn mainly exist as residual fractions, with U showing the highest residual proportion, followed by Cr and As—consistent with previous studies [45]—indicating low mobility and bioavailability under natural conditions. Notably, Cd exhibits the highest acid-soluble and reducible fractions, establishing it as a priority pollutant.

Sediments as predominantly occur as F1 fractions in all six zones, paralleled by substantial Ni, Zn fractions downstream, signaling high bioavailability. Cd shows elevated F2 fractions throughout, while Pb exhibits enhanced F2 fractions in Zones III-IV (abandoned mines, tailing areas, Fe, Mn-rich). U, Cu display prominent F3 fractions in Zone VI (organic-rich depositional environment). The Cd acid-soluble fraction significantly exceeds other elements, suggesting mining activities and polymetallic ore weathering drive its mobilization.

### 3.3. Mineralogy of Soil and Sediment

**Figure 4:**
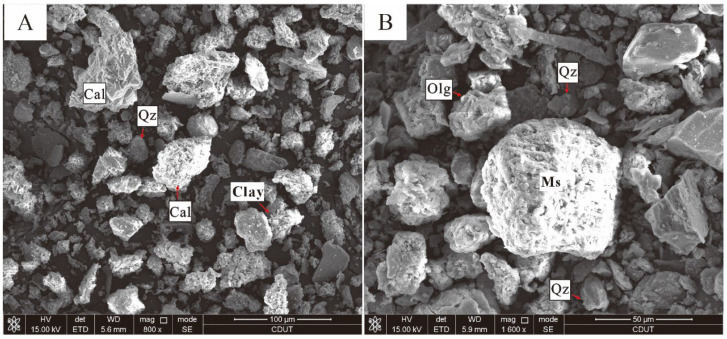
SEM analysis of sediment (**A**) and soil (**B**). Cal = Calcite; Qz = Quartz; Olg = Oligoclase; Ms = Muscovite.

The mineralogical profiles (Tables S1 and S2) reveal that soil and sediment matrices predominantly comprise feldspar, muscovite, quartz, clay minerals (9.7 ± 0.9%), calcite sphalerite, and bernalite. As shown in the elemental–mineral correlograms, calcium (R = 0.68, *p* < 0.01) and calcite (R = 0.72, *p* < 0.001) exhibit the strongest positive correlations with PTEs. Representative samples S4 and T25 (with highest PTE concentrations; Figure 4 SEM-EDS) contain elevated calcite. In alkaline soils, PTEs tend to form hydrated hydroxides and insoluble carbonates or become adsorbed and immobilized by soil colloids, providing a certain buffering effect. Calcite is an important binder for soil aggregates and can form humus with organic matter, increasing the soil’s surface area and negative charge, thereby enhancing the adsorption of Cr^3+^, Cu^2+^, Pb^2+^, Cd^2+^, and Zn^2+^. However, due to the hydrolysis effect in aquatic environments, the aggregates formed in sediments exhibit poor stability [46,47].

However, the mineral composition of soils and sediments predominantly consists of feldspar, quartz, and muscovite, which are chemically stable and exhibit limited correlation with heavy metal enrichment. For instance, samples with high quartz content (e.g., S01 and T11) may restrict heavy metal migration, due to their low porosity or poor permeability. Sample S30 (51.9% quartz + 41.9% feldspar) might display lower heavy metal concentrations if adsorptive minerals are absent.

S demonstrated a strong positive correlation with potentially toxic elements (PTEs). Sulfide minerals (e.g., sphalerite), iron oxides (e.g., bernalite), and clay minerals directly govern the occurrence forms and mobility of PTEs [48]. These reactive minerals can adsorb, precipitate, or stabilize metals (e.g., Zn, Cd, As, Pb) through chemical interactions, thereby controlling their environmental risks or resource potential. Samples S4 and T25, which have high heavy metal concentrations, were selected for SEM analysis in Figure 4. For example, sphalerite (ZnS) can oxidize through the following reaction, releasing Zn into the environment: ZnS(s) + 2O_2_(aq) → Zn^2+^ + SO_4_^2−^.

**Figure 5:**
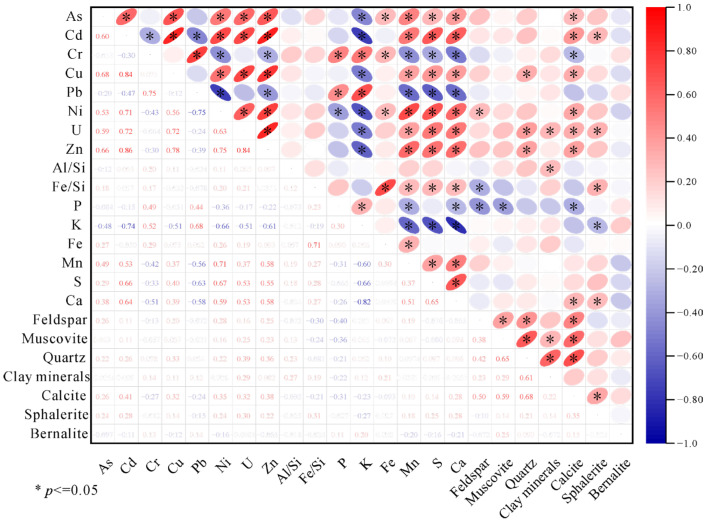
Correlation analysis of elemental compositions, element ratios, and mineralogical parameters in sediment and soil optimization and rationale.

**Figure 6:**
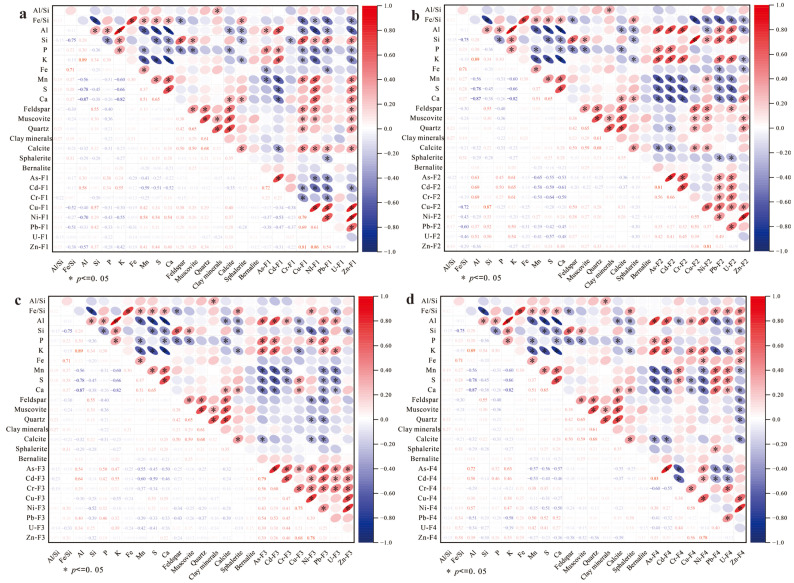
Inter-fraction correlations of BCR sequential extraction speciation with elemental associations and mineralogical phases. (**a**). Correlations between elements in F1 fraction and accessory minerals; (**b**). Correlations between elements in F1 fraction and accessory minerals; (**c**). Correlations between elements in F1 fraction and accessory minerals; (**d**). Correlations between elements in F4 fraction and accessory minerals. “*” indicates that the statistical result reaches significance at the 0.05 level.

In sediments and soils, as shown as Figure 5 and Figure 6, Mn, S, and Ca exhibited moderate correlations (r = 0.40–0.52*) with acid-soluble fractions (F1) of Ni, Cu, and Zn. Weak acids preferentially dissolve carbonate minerals, releasing Ca^2+^ and carbonate-bound metals (Ni, Cu, Zn) via congruent dissolution, resulting in source-dependent covariation between Ca and F1. In carbonate-rich zones, F1-Ca and F1 metals (Ni, Cu, Zn) often exhibit codependent trends, though deviations from linearity (r^2^ = 0.18–0.34) suggest contributions from non-carbonate phases (e.g., organic matter or sulfides). Manganese oxides, serving as key adsorbents for transition metals, partially dissolve under acidic conditions, liberating Mn^2+^ and adsorbed metal ions (e.g., Ni^2+^, Cu^2+^), thereby driving Mn-F1 metal correlations. Sulfur (as sulfate) may synchronize with Ca^2+^ and Mn^2+^ by ion exchange with SO_4_^2−^-bridged metal complexes.

As shown Figure 6b, silicate minerals (feldspar, muscovite, quartz) showed moderate positive correlations (r ~0.47–0.55) with reducible fractions (F2) of metals bound to Fe-Mn oxides. This linkage likely arises from Fe and Mn cycling, which weathering of silicates (e.g., biotite) releases Fe^2+^ and Mn^2+^, oxidizing to form secondary oxides (e.g., ferrihydrite, goethite). F2 fraction (e.g., As, Pb) subsequently adsorb or coprecipitate onto these neoformed oxides. Silicate grains (e.g., quartz) act as nucleation templates for Fe-Mn oxide coatings, generating cryptic adsorption sites undetectable by bulk XRD but critical for metal sequestration. Lithogenic oxidation regions with high quartz content (e.g., S3, S4) often exhibit concurrent feldspar weathering, amplifying Fe-driven oxide generation and F2-metal enrichment. No systematic correlations were observed between residual (F3), oxidizable (F4) metal fractions, and mineral phases.

### 3.4. Spatial Distribution Characters of PTEs

**Figure 7:**
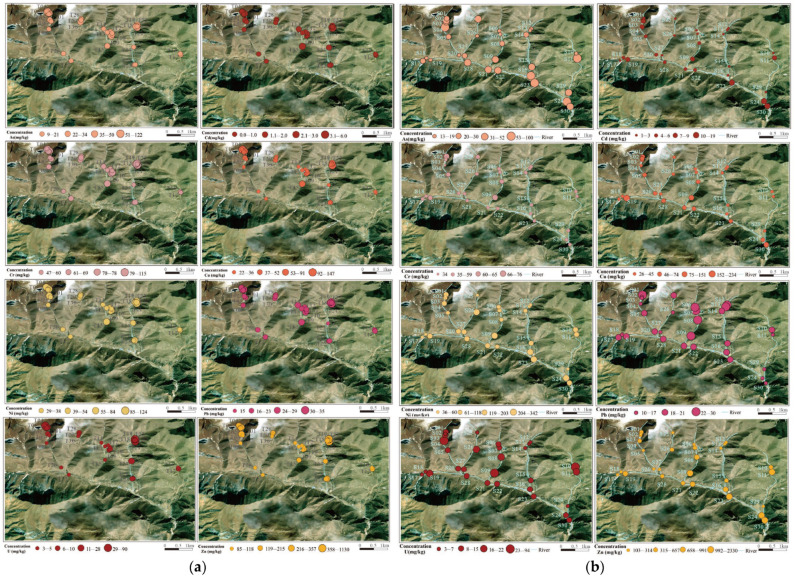
Spatial distribution of PTEs. (**a**) Soil; (**b**) sediment.

As shown in Figure 7, the distribution of As, Cd, Cr, Cu, Ni, Pb, Zn, and U in the surface soil of the study area is related to the distribution of uranium ore bodies or tailings ponds, with high-value areas mainly located at the T14 sampling point in the northwest of the mining area—on the ore-rich lithological zone—and at the T15 sampling point in the south of the mining area, as well as the T23 and T25 sampling points near the tailings pond. The average concentration of Pb is lower than the soil background value of Sichuan Province, and its distribution is relatively uniform, with a small coefficient of variation, likely influenced by geological background materials and weathering accumulation, with minimal impact from human activities. The higher heavy metal concentrations in the soil near the tailings pond may be due to the accumulation of tailings and the leaching of heavy metal ions into the soil by rainwater.

The spatial distribution of heavy metal concentrations in the sediments around the Zoige uranium mining area is shown in Figure 7b. The overall spatial distribution of heavy metals is characterized by the highest concentrations along the east–west trending ore bodies, downstream of the mining area and tailings pond, or at river confluences. The concentrations of Cr and Pb are lower than the soil background value of Sichuan Province, with small coefficients of variation, indicating minimal influence from human activities, and their distribution may be related to the natural geological background. The distribution of Cu differs from that of Cr and Pb, with higher concentrations at points S09, S19, and S30, possibly due to influences from human mining activities or other factors, in addition to the natural background. The highest concentration of Ni is found in Ⅵ, likely resulting from the deposition of Ni-bearing suspended particles carried by water flow from other gullies into the Sumulitanggou. The concentration of Zn increases downstream in Ⅲ, Ⅳ, and Ⅴ, while in Ⅵ higher Zn concentrations are observed at river confluences, such as at sampling points S23 at the confluence of Ⅳ and Ⅵ, and downstream points S24 and S30. The spatial distribution of U, particularly the high-concentration areas, is closely related to the east–west trend of the uranium ore bodies. For example, sampling points S04, S26, S08, and S10 from east to west are located in high-concentration zones of U within their respective gullies. Additionally, the U concentration at point S09 is 55 mg/kg, likely due to U from the open-pit mining area in Ⅲ being leached by rainwater and transported downstream by flowing water, where it is subsequently deposited.

### 3.5. Source Apportionment by Positive Matrix Factorization 

Correlation analysis (Figure 5) revealed distinct geochemical groupings among PTEs: Ni-Zn (r^2^ = 0.92), Ni-Cd (r^2^ = 0.87), U-Zn (r^2^ = 0.86), Cd-Cu (r^2^ = 0.85), Cd-Zn (r^2^ = 0.84), and Cu-Ni (r^2^ = 0.83) exhibited congeneric behavior, suggesting a shared lithogenic or anthropogenic source (e.g., uranium ore processing or sulfide weathering). As showed moderate correlations with U and six other metals (Cd, Cr, Cu, Ni, Zn), while U correlated similarly (r^2^ = 0.5–0.7) with As, Cd, Cr, Cu, Ni, and Zn. This pattern implies potential redox-driven coprecipitation or joint mobilization via organic complexes. Pb demonstrated weak correlations (r^2^ < 0.4) with Cd, Ni, U, and Zn, and Cr exhibited limited associations (r^2^ < 0.40) with other metals. Their geochemical decoupling likely reflects distinct sources, such as Pb, atmospheric deposition from legacy leaded fuels or isolated mineral phases.

Intermediate correlations were observed for Ni-Zn (r^2^ = 0.81), Cd-Cu (r^2^ = 0.8), Cu-Zn (r^2^ = 0.73), U-Zn (r^2^ = 0.68), U-Ni (r^2^ = 0.65), Cd-Zn (r^2^ = 0.64), Cr-Pb (r^2^ = 0.63), Cu-Ni (r^2^ = 0.63), and As-Ni (r^2^ = 0.61), indicative of overlapping transport pathways. Cd-Pb showed significant anticorrelation, likely due to competitive adsorption on clay minerals or phase-specific sequestration.

For enhanced source discrimination, data were processed using EPA PMF 5.0 (a US EPA-developed Positive Matrix Factorization program). Repeated runs were performed by setting 3–5 factors. The optimal number of factors was determined when Qrobust/Qtrue exhibited a sharp decline, with increasing factor numbers.

**Figure 8:**
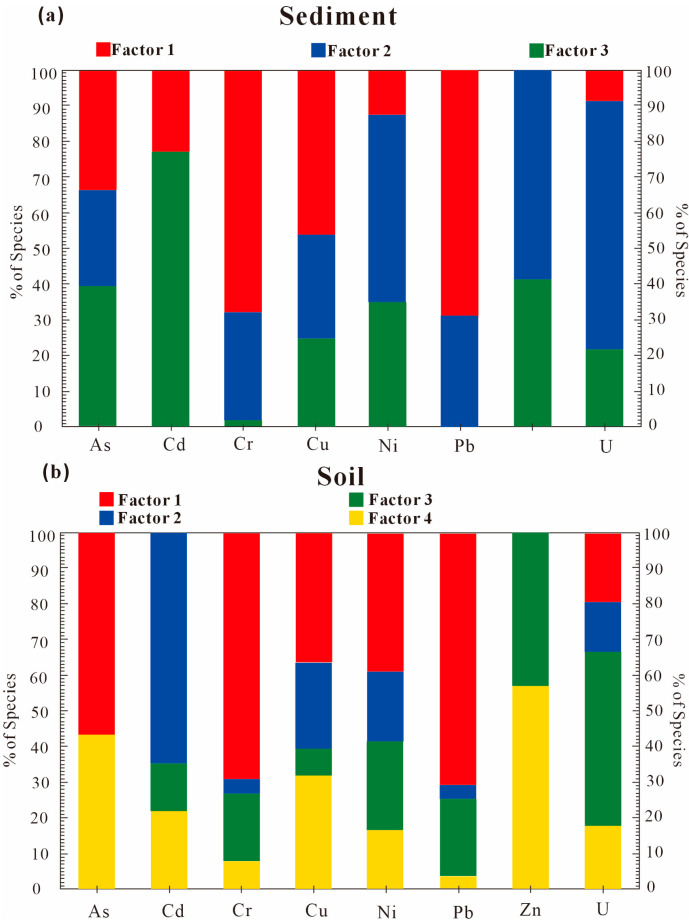
Contribution of different factors to heavy metal pollution in sediment (**a**) and soil (**b**).

Factor 1 is a natural lithogenic source in Figure 8, with Pb (71%) > Cr (69%) > As (57%) > Ni (40%) > Cu (37%), Cr and Pb, showing highly significant correlation. Cr and Pb concentrations are below Sichuan soil background values, with low coefficients of variation (Cr: 0.18; Pb: 0.15). I_geo_ < 0 and BCR sequential extraction results reveal >80% of Cr, Pb reside in residual fractions (silicate-bound), indicating minimal anthropogenic influence.

Factor 2 is an anthropogenic–natural composite source. Ranked in descending order of contribution loadings are the following: Cd (65%) > Cu (24%) > Ni (20%) > Zn (13%). Cd, with mean content higher than the Sichuan background, Cd-Cu (r = 0.85, *p* < 0.01), and Cd-Zn (r = 0.84, *p* < 0.01), have strong pairwise correlations. Localized enrichment in Zone IV (ore processing/tailing areas) is linked to sulfidic mineral smelting and uranium leaching.

Factor 3 is mining-related pollution. Ranked in descending order of contribution loadings are Zn (49%) > U (43%) > Ni (25%) > Pb (22%). U and Zn (coefficients of variation: 1.5 and 1.0, respectively) and elevated concentrations coincide with smelters and active mining zones.

Factor 4 is Uranium–As geogenic activity. Ranked in descending order of contribution loadings are U (57%) > As (43%) > Cu (32%). U-As co-mobilization via uranyl arsenate complexes (e.g., autunite/metatorbernite precipitation) is triggered by weathering of uraniferous tailings. Cement-sealed, yet unmanaged, waste ores act as persistent U and As sources during rainfall leaching.

In fluvial sediments, Factor 1 is the Lithogenic background. Ranked in descending order of contribution loadings are Pb (69%) > Cr (68%) > Cu (46%) > As (33%). Low variability (Cr: CV = 0.13; Pb: CV = 0.16) and sub-background concentrations align with chalcophile/siderophile-element behavior in erosional regimes [49].

Factor 2 presented elevated loadings for U (70%), Zn (59%), and Ni (53%), where As, Cr, Cu, and Pb showed secondary loadings. Notably, sediment uranium concentrations exceeded the Chinese soil background value 5.57-fold (CV = 1.15), indicating substantial anthropogenic inputs. Spatial mapping revealed that U hotspots (Figure S1) align linearly with east–west trending uranium ore belts (Mann–Whitney U = 187, *p* < 0.001). We attribute this pattern to the long-term leaching of U-bearing acid mine drainage (AMD) from adjacent mining operations, consistent with prior findings in uranium-enriched catchments.

Factor 3 was characterized by significant loadings of Cd (77%), Zn (41%), As (40%), and Ni (35%). The strong Cd-Zn covariation (r = 0.89, *p* < 0.001) parallels their co-occurrence in vehicular lubricants and tire additives. Concurrently, As-Ni-Zn anomalies corresponded spatially with legacy tailings ponds (Kruskal–Wallis H = 23.7, *p* < 0.01), implicating combined mechanisms: (1) atmospheric deposition from vehicle emissions; (2) leachate infiltration from mining waste reservoirs. Factor 3, therefore, represents a mixed source involving mining transport activities and weathering of metallogenic parent materials.

### 3.6. Risk Assessment of PTEs

#### 3.6.1. Geo-Accumulation Index

**Figure 9:**
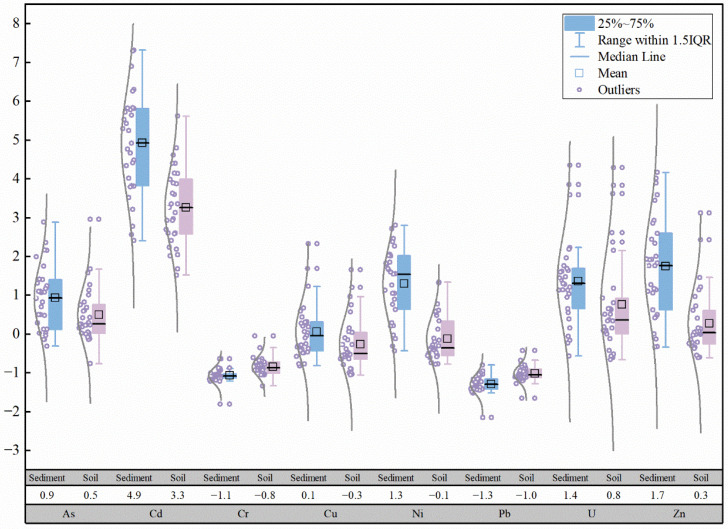
Geo-accumulation index of PTEs in surface soils and sediments. The shade represents types and elements respectively, while the white column indicates mean values. Blue symbols represent sediment data, while purple represents soil data.

The geo-accumulation index indicates that, except for Cr and Pb, the elements As, Cd, Cu, Ni, Zn, and U in the soil and sediments exhibit varying degrees of pollution in Figure 9. Statistical analysis of the geo-accumulation index evaluation results is provided in the Supplementary Materials (Tables S1–S16). Overall, Cd in the soil is classified as heavily polluted, with 53.33% of the soil samples showing strong or higher levels of Cd pollution, and the geo-accumulation index ranging from 1.52 to 5.62. U in the soil is generally classified as lightly to moderately polluted, with the geo-accumulation index ranging from −0.66 to 4.29, spanning pollution levels from non-polluted to strong–extremely-severe pollution. Among these, 20% of the samples are non-polluted, 53.33% are lightly to moderately polluted, and a few samples reach strong to extremely severe pollution levels. This is mainly due to the proximity of samples T14, T15, and T25 to the tailings pond and mining area, resulting in relatively higher pollution levels. The overall pollution level of As in the soil is classified as light to moderate, with the geo-accumulation index ranging from −0.76 to 2.96, spanning pollution levels from non-polluted to moderate–strong pollution. Among these, 60% of the samples are lightly to moderately polluted, 20% are non-polluted, and a few samples reach moderate or moderate–strong pollution levels, mainly due to their proximity to the mining area and tailings pond. The overall pollution level of Zn in the soil is relatively weak, with the geo-accumulation index ranging from −0.16 to 3.12, spanning pollution levels from non-polluted to moderate–strong pollution. Among these, 50% of the samples are non-polluted, and 33% are lightly to moderately polluted. The pollution levels of Cu and Ni in the soil are relatively weak, with a high proportion of non-polluted samples, accounting for 73.33% and 70% of the samples, respectively.

In the sediments, Cd is, overall, classified as extremely severe pollution, with the geo-accumulation index ranging from 2.41 to 7.32, spanning pollution levels from moderate–strong pollution to extremely severe pollution. Among the samples in the study area, 90% of the sediment samples show strong or higher levels of Cd pollution. U in the sediments is generally classified as moderate pollution, with the geo-accumulation index ranging from −0.56 to 4.35, spanning pollution levels from non-polluted to strong–extremely severe pollution. Among these, 53% are moderately polluted, 17% are lightly to moderately polluted, 20% are strongly or extremely severely polluted, and 10% are non-polluted. As in the sediments is generally classified as light–moderate pollution and moderate pollution, with the geo-accumulation index ranging from −0.31 to 2.89, spanning pollution levels from non-polluted to moderate–strong pollution. Among these, 37% are lightly to moderately polluted, 37% are moderately polluted, and 13% are non-polluted or moderately-strongly polluted. In the sediments, Zn is overall classified as moderate pollution, with the geo-accumulation index ranging from −0.33 to 4.17, spanning pollution levels from non-polluted to strong–extremely severe pollution. Among these, 37% of the samples are moderately polluted, and 37% are moderately-strongly or more severely polluted. Ni in the sediments is generally classified as moderate pollution, with the geo-accumulation index ranging from −0.43 to 2.81, spanning pollution levels from non-polluted to moderate–strong pollution. Among these, 40% are moderately polluted, 27% are moderately-strongly polluted, and 13% and 20% are non-polluted and lightly to moderately polluted, respectively. The pollution level of Cu in the soil is relatively weak, with a high proportion of non-polluted samples at 53%, lightly to moderately polluted samples at 37%, and moderately polluted and moderately-strongly polluted samples at 7% and 3%, respectively.

#### 3.6.2. The Contamination Factor

**Figure 10:**
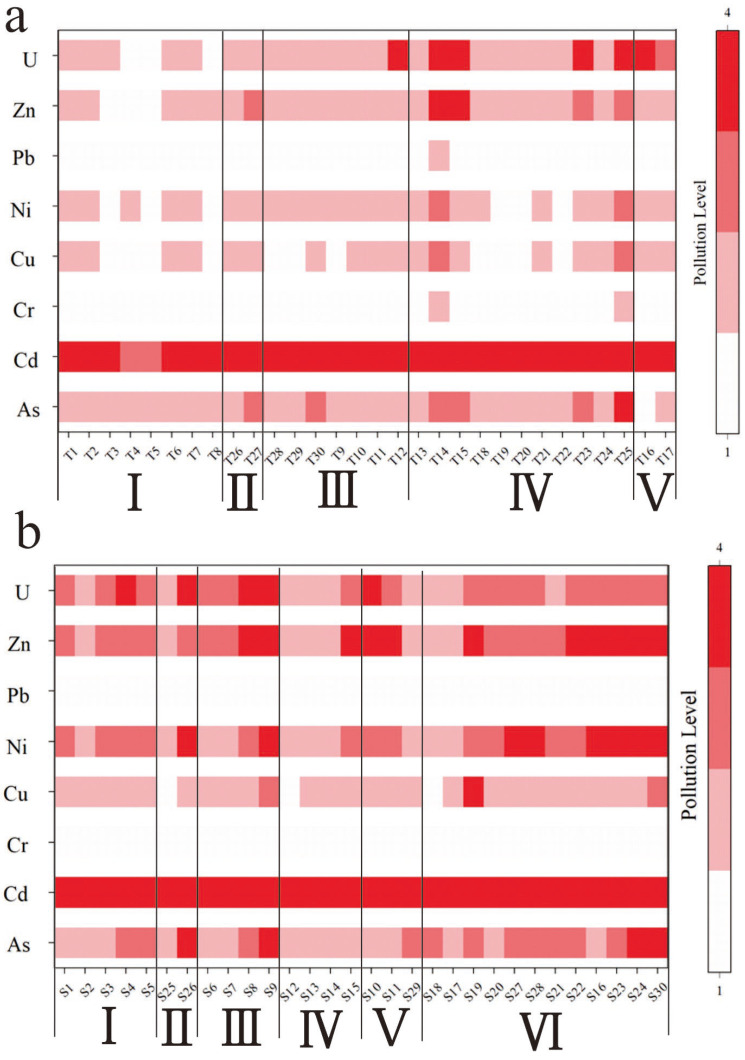
Classification of heavy metal pollution factors. (**a**) Soils; (**b**) sediments.

The CF of Cd in the surface soil of the study area is the highest, followed by U, As, and Zn. The PLI results for the surface soil in the study area are shown in Figure 10a. The PLI for sample T14, located in the northwest of the mining area, is 6.01, while the PLI for samples T25 and T23, located south and north of the tailings pond, are 5.79 and 3.29, respectively, indicating extremely strong pollution. Most of the remaining samples are moderately to strongly polluted. The PLI values for samples T14, T15, T23, and T25 in the study area are significantly higher, reaching extremely strong pollution levels, primarily due to their proximity to the open-pit mining area and tailings pond.

In the sediments, the CF of Cd is the highest, followed by U, As, and Zn, while Cr and Pb have the lowest CF. The PLI results for the sediments in the study area are shown in Figure 10. The PLI for sample S09 is 7.63, followed by samples S23 and S24 with PLI values of 5.38 and 5.52, respectively. The PLI values for samples S09, S23, and S24 in the study area are significantly higher, reaching extremely strong pollution levels, likely due to their proximity to the open-pit mining area and downstream regions of the tailings pond. When rivers flow through pollution sources, PTEs are adsorbed into the sediments, and the sediments carrying PTEs are deposited downstream, due to gravity, resulting in pollution in the downstream areas of the pollution sources.

The calculated comprehensive PLI for the surface soil in the study area is 1.78, indicating an overall moderate pollution level. This suggests that the soil environmental quality in the study area is significantly influenced by human activities, in addition to the natural geological background. The PLI levels of the 30 sampling points in the area were classified, and the results are shown in Table 8. Among these, 70% of the samples are moderately polluted, 16.7% are strongly polluted, and 13.3% are extremely strongly polluted. The comprehensive PLI for the sediments is 2.99, indicating an overall strong pollution level. The PLI levels of all sampling points were classified and statistically analyzed, and the results are shown in Table 9. Among these, 20% of the samples are moderately polluted, 30% are strongly polluted, and 50% are extremely strongly polluted.

#### 3.6.3. Potential Ecological Risk

The PERI method was used to assess the pollution levels of eight PTEs in the soil and sediment, and the evaluation results are shown in Table 9 and Table 10. The average single potential ecological risk indices of the eight PTEs in the study area are ranked as Cd > U > As > Ni > Cu > Pb > Zn > Cr. Among these, the single-factor potential ecological risk indices of Cr, Cu, Ni, Pb, and Zn are all less than 40, indicating a slight risk level. The main potential ecological risk factors in the study area are As, Cd, and U. This is partly due to their high toxicity coefficients, which are 10, 30, and 20, respectively, contributing significantly to the single PERI. On the other hand, it is due to the varying degrees of enrichment of these three PTEs in the study area. The comprehensive PERI ranges from 180 to 2746, with an average of 684. Strong risk accounts for 36.7%, moderate and very strong risk account for 23.3% and 26.7%, respectively, and extremely strong risk accounts for 13.3%, indicating an overall high ecological risk.

The average single potential ecological risk indices of the eight PTEs in the sediments of the study area are ranked as Cd > U > As > Ni > Cu > Zn > Pb > Cr. Among these, the single-factor potential ecological risk indices of Cr, Cu, Pb, and Zn are all less than 40, indicating a slight risk level. The main potential ecological risk factor in the study area is Cd, followed by U and As. The comprehensive PERI ranges from 294 to 7408, with an average of 2183. Extremely strong risk accounts for 53.3%, very strong risk accounts for 23.3%, and moderate and strong risk account for 3.3% and 10%, respectively. The overall ecological risk is high, and should be given significant attention.

#### 3.6.4. Potential Human Health Risk

**Figure 11:**
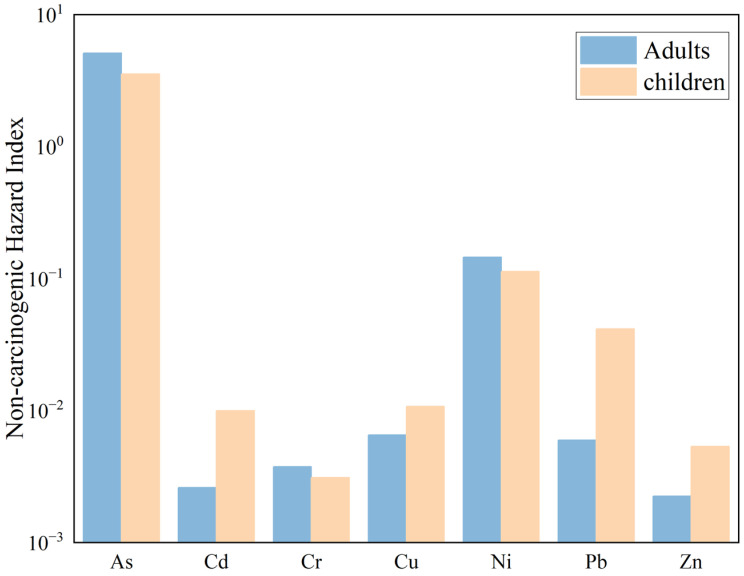
Results of non-carcinogenic health risk assessment.

A non-carcinogenic health risk assessment was conducted for PTEs in the surface soil of the Zoige uranium mining area under three exposure pathways, and the results are shown in Figure 11. The single non-carcinogenic health risk indices (HQ) for adults exposed to PTEs through oral ingestion, dermal contact, and inhalation are as follows: As (5.11 × 10^0^), Cd (2.61 × 10^−3^), Cr (3.76 × 10^−3^), Cu (6.50 × 10^−3^), Ni (1.45 × 10^−1^), Pb (5.94 × 10^−3^), and Zn (2.24 × 10^−3^). Except for As, for which the HQ value is greater than 1, the HQ values of the other PTEs are all less than 1, indicating that As poses a certain potential hazard to adults. The total non-carcinogenic risk index (HI) for adults is 5.27 × 10^0^, and since HI is greater than 1, it indicates that the exposure exceeds the threshold, suggesting a relatively high non-carcinogenic risk that requires attention. Overall, the PTEs in the soil of the Zoige uranium mining area pose a high non-carcinogenic risk to adults.

The single non-carcinogenic health risk indices (HQ) for children exposed to PTEs through oral ingestion, dermal contact, and inhalation are as follows: As (3.52 × 10^0^), Cd (9.93 × 10^−3^), Cr (3.11 × 10^−3^), Cu (1.07 × 10^−2^), Ni (1.13 × 10^−1^), Pb (4.14 × 10^−2^), and Zn (5.23 × 10^−3^). Among these, the HQ value for As is greater than 1, indicating that As also poses a non-carcinogenic risk to children. The total non-carcinogenic risk index (HI) for children is 3.72 × 10^0^, and since HI is greater than 1, it indicates that the exposure exceeds the threshold, suggesting a relatively high non-carcinogenic risk. Therefore, the PTEs in the soil of the Zoige uranium mining area also pose a high non-carcinogenic risk to children.

**Figure 12:**
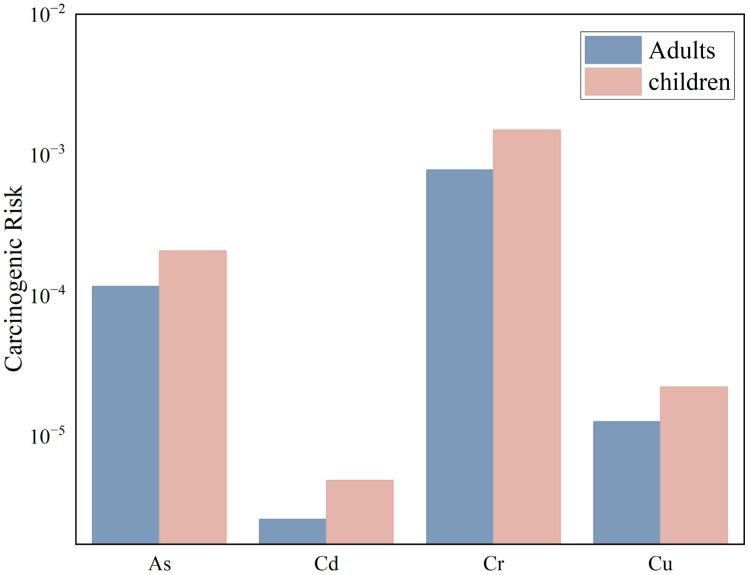
Results of carcinogenic health risk assessment.

Due to the lack of carcinogenic slope factors for Cu, Pb, Zn, and U, this study only conducted a carcinogenic health risk assessment for the following four PTEs: As, Cd, Cr, and Ni. The carcinogenic health risk assessment for PTEs in the surface soil of the Zoige uranium mining area under three exposure pathways is shown in Figure 12.

For adults exposed through oral ingestion, dermal contact, and inhalation, the carcinogenic health risk indices (CR) for the PTEs As, Cd, Cr, and Ni are 1.15 × 10^−4^, 2.55 × 10^−6^, 7.81 × 10^−4^, and 1.26 × 10^−5^, respectively, with the order of risk being Cr > As > Ni > Cd. The CR values for As and Cr exceed 1.0 × 10^−4^, indicating that As and Cr in the soil of the study area pose significant carcinogenic risks to adults. The total carcinogenic health risk (TCR) for adults is 9.12 × 10^−4^, and since TCR > 1 × 10^−4^, it indicates that adults in the study area face a high carcinogenic risk.

For children exposed through oral ingestion, dermal contact, and inhalation, the carcinogenic health risk indices (CR) for the PTEs As, Cd, Cr, and Ni are 2.07 × 10^−4^, 4.84 × 10^−6^, 1.50 × 10^−3^, and 2.23 × 10^−5^, respectively, with the order of risk being Cr > As > Ni > Cd. The CR values for As and Cr exceed 1.0 × 10^−4^, indicating that As and Cr in the soil of the study area pose significant carcinogenic risks to adults. The total carcinogenic health risk (TCR) for children is 1.74 × 10^−3^, and since TCR > 1 × 10^−4^, it indicates that children in the study area also face a high carcinogenic risk.

## 4. Conclusions

Through a comprehensive integration of the PMF model, geochemical indices, and health risk assessment, this study elucidates the sources and ecological impacts of heavy metal contamination in surface soils and sediments of the Zoige uranium mining area. Key findings reveal the following:

(1) Cd emerged as the primary pollutant of concern, exhibiting the highest bioavailability due to its acid-soluble and reducible fractions. Acid-soluble and reducible fractions of Ni, Cu, Zn, As, and Pb are governed by carbonate dissolution and Fe-Mn oxide dynamics via silicate weathering, while residual (F3) and oxidizable (F4) fractions show weak mineral-phase dependencies.

(2) Spatial Distribution Patterns: Cr-Ni (soil) and Cr-Pb (sediments) share spatial homogeneity, reflecting natural geological backgrounds; As, Cd, and Cu distributions align with uranium ore bodies, while U enrichment correlates with ore bodies and tailings ponds. Downstream sediment hotspots suggest fluvial transport influence; dominant silicate mineralogy retains PTEs in residual phases, yet bioavailable Cd poses significant ecological risks.

(3) PTE hotspots clustered near uranium ore belts, tailings ponds (e.g., T14, T25), and downstream confluences, aligning with mining activities and hydrologic transport. Factor analysis delineated four distinct sources. Natural lithogenic inputs (Cr, Pb, As) with residual dominance and minimal anthropogenic influence as confirmed by. Anthropogenic–natural composites (Cd, Cu, Ni, Zn) were linked to ore processing and sulfide weathering. Mining-related pollution (U, Zn, Pb) was driven by smelting and AMD leaching. In fluvial sediments, Cd-Zn-As anomalies correlated with vehicular emissions and legacy tailings, while uranium enrichment was attributed. 

(4) Pollution Severity: in the soil, geo-accumulation indices rank pollution as Cd (3.25, extreme) > U (0.89) > As (0.49) > Zn (0.27) > Cu, Cr, Pb (unpolluted), with a moderate overall pollution load (PLI = 1.78); in sediments, Cd (4.92, extreme) dominates, followed by Zn (1.75), U (1.49), and Ni (1.3), causing severe cumulative pollution (PLI = 2.99). Health risks (USEPA) exceed safety thresholds, with higher carcinogenic, non-carcinogenic risks for adults than children.

These findings underscore the dual impact of natural geology and anthropogenic activities, emphasizing urgent remediation needs for Cd and U hotspots to mitigate ecological and public health threats.

## Figures and Tables

**Table 1:** Improved BCR sequential extraction method.

Fraction ID	Extractant	Process	Speciation of PTEs
F1	Add 20 mL 0.11 mol/L (pH = 2) CH_3_COOH	Conduct continuous agitation at 25 ± 0.5 °C for 16 h.	Acid extractable state
F2	Add 20 mL 0.5 mol/L (pH = 2) NH_3_OHCl	React with 30% H_2_O_2_ at RT (25 °C) for 1 h	Reducible state
F3	Add 20 mL 8.8 mol/L (pH = 2) H_2_O_2_ And 1.0 mol/L (pH = 2) CH_3_COONH_4_	Evaporate to dryness via stepwise water baths (85 °C → 95 °C). Add 2 mol/L CH_3_COONH_4_ solution, agitate at RT for 16 h	Oxidizable state
F4	Add 10 mL H_2_NO_3_, 10 mL HF And 3 mL HClO_4_	Treat with concentrated HNO_3_ (68%), incubate overnight at RT. Heat to 120 °C on a hotplate (caution: acid fumes). Sequentially add HF (40%) and HClO_4_ (70%) until achieving near-dry viscous residue	Residual state

**Table 2:** Classification of geo-accumulation index (Igeo) in relation to soil quality.

I_geo_	Soil Quality	I_geo_ Classes
I_geo_ ≤ 0	Unpolluted	0
0 < I_geo_ ≤ 1	Unpolluted to moderately polluted	1
1 < I_geo_ ≤ 2	Moderately polluted	2
2 < I_geo_ ≤ 3	Moderately polluted to highly polluted	3
3 < I_geo_ ≤ 4	Highly polluted	4
4 < I_geo_ ≤ 5	Highly polluted to very highly polluted	5
5 < I_geo_	Very highly polluted	6

**Table 3:** The classification criteria of CF and PLI.

*CF*	Pollution Levels	*PLI*	Pollution Levels
*CF* ≤ 1	No pollution	*PLI* ≤ 1	Baseline pollution
1 < *CF* ≤ 3	Low pollution	1 < *PLI* ≤ 2	Moderate pollution
3 < *CF* ≤ 6	Moderate pollution	2 < *PLI* ≤ 3	Significant pollution
*CF* ≥ 6	High pollution	*PLI* ≥ 3	Extremely high pollution

**Table 4:** Classification standard for potential ecological risks of PTEs.

*E_i_*	*RI*	Potential Risk
*E_i_* ≤ 40	*RI* ≤ 150	Low risk
40 ≤ *E_i_* ≤ 80	150 ≤ *RI* ≤ 300	Moderate risk
80 ≤ *E_i_*≤ 160	300 ≤ *RI* ≤ 600	Considerable risk
160 ≤ *E_i_* ≤ 320	600 ≤ *RI* ≤ 1200	High risk
*E_i_* ≥ 320	*RI* ≥ 1200	Very high risk

**Table 5:** Exposure parameters related to health risk assessment model.

Parameters	Meaning	Value		Reference
		Adults	Children	
*CS*	Pollutant concentration of soil (mg/kg)	—	—	This Study
*IR*	Soil ingestion rate (mg/d)	100	200	[25]
*AF*	skin adhesion factor (kg/cm^2^)	0.2	0.2	[26]
*TF*	Conversion factor (kg/mg)	1 × 10^−6^	1 × 10^−6^	
*EF*	Exposure frequency (d/a)	180	180	[25]
*ED*	Exposure duration (a)	24	6	
*BW*	The average body weight (kg)	62	15.9	
*AT*	Average exposure time (d)	ED×365 (non-carcinogenic), 70 × 365 (carcinogenic)		
*SA*	Exposure of the skin surface area (cm^2^)	1.6 × 10^4^	2800	
*ABS*	Skin absorption factor	As: 0.03, Cu: 0.06, Pb: 0.006, Zn: 0.02, Cd: 0.001, Ni: 0.091, Cr: 0.04		[27]
*IAR*	Inhalation rate(m^3^/d)	16.1	8.3	[28]
*PEF*	Particle emission factor (m^3^/kg)	1.36 × 10^9^	1.36 × 10^9^	

Footer: — means no data.

**Table 6:** Descriptive statistics of heavy metal content in surface soil and sediment samples (mg/kg).

	Metals	pH	As	Cd	Cr	Cu	Ni	Pb	Zn	U
Soil	Max	7.9	122	5.8	115	147	124	34.5	1130	89.7
	Min	6.18	9.2	0.34	47	22.4	28.5	14.7	85	2.9
	Average	7.03	25.6	1.44	67.1	44.2	49.5	23.2	201	13.8
	Median	7.06	18.7	1.09	65	32.8	38.2	22.3	131	5.85
	SD	0.45	20.1	1.13	12.3	27.9	24.9	3.43	210	20.3
	CV	0.06	0.78	0.78	0.18	0.63	0.5	0.15	1.04	1.47
	BGV	—	10.4	0.08	79	31.1	32.6	30.9	86.5	3.05
	Average/BGV	—	2.46	18	0.85	1.42	1.52	0.75	2.33	4.53
	RSV	—	30	0.3	200	100	100	120	200	—
Sed	Max	8.02	116	19	76	234	342	26.7	2330	93.5
	Min	6.83	12.6	0.63	34	26.5	36.3	10.4	103	3.1
	Average	7.7	35.5	5.25	56.9	56.5	143.6	19.2	646	16.9
	Median	7.77	31.1	4.05	56	44.5	143	18.6	452	11.2
	SD	0.27	23.2	4.71	7.34	41.5	81.7	3.01	608	19.5
	CV	0.04	0.65	0.9	0.13	0.73	0.57	0.16	0.94	1.15
	BGV	—	10.4	0.08	79	31.1	32.6	30.9	86.5	3.05
	Average/BVG	—	3.41	65.6	0.72	1.82	4.4	0.62	7.47	5.53
	RSV	—	30	0.3	200	100	100	120	200	—
Rock	Average	—	108	21.46	53.67	145.25	105.3	22.3	2434	7936

Footer: S (soil, *n* = 30); Sed (sediment, *n* = 30; SD: standard deviation; CV: coefficient of variation); BGV: background value; RSV: risk screening value for agricultural soil pollution in China; bedrock (*n* = 10).

**Table 7:** PTEs concentrations in surface soils and sediments from selected uranium mining areas in China (mg/kg).

	Uranium Mining Area	As	Cd	Cr	Cu	Ni	Pb	Zn	U	Reference
soil	Zoige	25.6	1.4	67.1	44.2	49.5	23.2	201	13.8	Study Area
	Xiazhuang	3.6	0.13	15.8	10.5	7.4	90.6	71.7	34.7	[32]
	Bayanwula	1.1	0.009	14.6	4.8	5.4	73.6	18.1	—	[33]
	South China	17.6	—	71.9	—	—	31.1	—	13.7	[34]
	Nuheting	8.7	0.1	74.0	23.3	24.9	23.0	3.0	66.7	[35]
	East China	4.8	0.2	—	7.2	6.1	18.2	35.5	—	[36]
	Northern Guangdong	936	8.2	—	23.9	26.6	70.5	154	33.4	[37]
	Northwest China	135	0.3	29.8	—	—	21.2	258	—	[38]
	K_1_	2.5	18.2	0.9	1.4	1.5	0.8	2.3	4.5	—
	K_2_	2.3	14.8	1.1	2.0	1.8	0.9	2.7	4.6	—
Sed	Zoige	35.5	5.3	56.9	56.5	144	19.2	646	16.9	Study Area
	East China	8.6	0.4	33	17.1	12.8	35.4	68.1	—	[39]
	Beishanhe	—	0.3	88.5	6.2	300	41.4	13.7	—	[40]
	Jiangxi	—	1.6	0.5	4.9	—	1.9	—	3.3	[41]
	K_1_	3.4	66.5	0.7	1.8	4.4	0.6	7.5	5.5	
	K_2_	3.2	54.1	0.9	2.5	5.3	0.7	8.7	5.6	
	K_3_	3.9	41.7	1.1	2.8	6.2	0.8	9.7	7	
	Soil Background Values of Sichuan Province	10.4	0.1	79	31.1	32.6	30.9	86.5	3.1	
	Soil Background Values of China	11.2	0.1	61	22.6	26.9	26.0	74.2	3	
	Sediment Background Values of China	9	0.126	54	20	23	23	67	2.4	[42]

Footer: — means no data; heavy metal concentrations are reported in mg/kg (parts per million); K_1_ and K_2_ are dimensionless coefficients (unitless). K_1_ represents the ratio of heavy metal concentrations in surface soils of the study area to the background values of Sichuan Province soils; K_2_ denotes the ratio of heavy metal concentrations in surface soils of the study area to the national background values of Chinese soils; K_3_ indicates the ratio of heavy metal concentrations in surface soils of the study area to China’s soil pollution risk screening values.

**Table 8:** Evaluation and statistics of pollution-load index in surface soils and sediments.

Sample		Baseline Pollution	Moderate Pollution	Significant Pollution	Extremely High Pollution	Total
Soils	Number	0	21	5	4	30
	Proportion (%)	0	70	16.7	13.3	100
Sediments	Number	0	6	9	15	30
	Proportion (%)	0	20	30	50	100

**Table 9:** Classification statistics of PERI of PTEs in surface soil.

Metals		Range	Average	Proportion of Potential Ecological Risk Levels %				
				Low	Moderate	Considerable	High	Very High
*E_i_*	As	8.85~117	24.6	90%	6.7%	3.3%	—	—
	Cd	129~2214	547	—	—	6.7%	30%	63.3%
	Cr	1.19~2.91	1.7	100%	—	—	—	—
	Cu	3.6~23.6	7.11	100%	—	—	—	—
	Ni	4.37~19	7.58	100%	—	—	—	—
	Pb	2.38~5.58	3.75	100%	—	—	—	—
	Zn	0.98~13.1	2.33	100%	—	—	—	—
	U	19~588	90.7	53.7%	27%	7%	37%	10%
*RI*		180~2746.	6847	—	23.7%	37%	27%	13.7%

**Table 10:** Classification statistics of PERI of PTEs in sediments.

Metals		Range	Average	Proportion of Potential Ecological Risk Levels %				
				Low	Moderate	Considerable	High	Very High
*E_i_*	As	12~111	34.1	76.7%	20%	3.3%	—	—
	Cd	239~7196	1995	—	—	—	10%	90%
	Cr	0.86~1.92	1.44	100%	—	—	—	—
	Cu	4.26~37.6	9.09	100%	—	—	—	—
	Ni	5.57~52.5	22	90%	10%	—	—	—
	Pb	1.68~4.32	3.10	100%	—	—	—	—
	Zn	1.19~26.9	7.47	100%	—	—	—	—
	U	20.3~613	111	20%	36.7%	33.7%	—	10%
*RI*		294~7408	2183	—	37.7%	10%	23.8%	53.7%

## Data Availability

The data presented in this study are available in the article.

## Supplementary Materials

The following supporting information can be downloaded at: https://www.mdpi.com/article/10.3390/toxics13070561/s1, Figure S1: Schematic geology uranium deposit site points at the Zoige; Table S1: Bedrock elemental content (mg/kg); Table S2: Element content (mg/kg) and Mineral content(%); Table S3: Element content (mg/kg) and Mmineral content (%); Table S4: Elemental content in sediments and soils by BCR sequential extraction (mg/kg); Table S5: Elemental content in sediments and soils by BCR sequential extraction (mg/kg); Table S6: Geoaccumulation index of sediments and soils; Table S7: Statistical analysis of geoaccumulation index evaluation results for surface soils; Table S8: Statistical analysis of geoaccumulation index evaluation results for Sediments; Table S9: Evaluation results of pollution load index in surface soils of the study area.; Table S10: Statistical evaluation of pollution load index in surface soils of the study area; Table S11: Evaluation results of pollution load index in sediment of the study area; Table S12: Statistical evaluation of pollution load index in sediments of the study area; Table S13: Exposure parameters related to health risk assessment model; Table S14: RfD and SF values of heavy metals in health risk assessment; Table S15: Non-carcinogenic health risk assessment results; Table S16: Carcinogenic health risk assessment results.
