# Supplementary material for: Geochemical Characteristics and Risk Assessment of PTEs in the Supergene Environment of the Former Zoige Uranium Mine
Source: Toxics. 2025 Jun 30;13(7):561. doi: 10.3390/toxics13070561 (PMC12300019; doi:10.3390/toxics13070561)
Supplement: Supplementary file 1 [file toxics-13-00561-s001.zip › toxics-3608023-supplementary.pdf]

Supplementary general situation of regional geology

Uranium mineralization exhibits layer control structural characteristics, and ores occur in the hanging wall of bedding fracture zones, concentrated at the intersection of folds and faults (FIGURE 1a). The combined thickness of the Cambrian and Ordovician strata in the mining area is 927 m, with uranium content in the rocks ranging from (8-12) ug/g. The thickness of the Silurian strata is greater than 1,147.3m, with a uranium content of (5-15) ug/g. Both are characterized by typical carbon-siliceous mudstone and local siliceous rock layers show uranium mineralization. The exposed stratigraphy within the mining zone primarily consists of Silurian and Quaternary strata, with the main ore-bearing layer being the upper section of the Yangchanggou Formation (S_1y^1) from the Lower Silurian System. In addition, the strata also incorporate the Yangchanggou Formation's lower section (S_1y^1), alongside the lower (S_1t^1) and upper (S_1t^2) sections of the Tal Formation from the Lower Silurian System. The distribution of strata in the zone mainly covers an east-west direction, presenting a strip-like configuration. Following a sequence from ancient to recent, the regional strata comprise:

(1) Lower Section of the Yangchanggou Formation (Lower Silurian System) (S_1y^1): Characterized by a gray to gray-black hue, the principal rock type in this stratum is medium-thick, shallowly-metamorphosed siltstone.

(2) The Upper Yangchanggou Formation (S_1y^2) of the Lower Silurian System represents the primary uranium deposit in the study area. Notably, uranium mineralization occurs within carbonaceous siliceous limestone and siliceous rock. Further, the stratum's lithology encompasses limestone, siliceous limestone, and carbonaceous silty slate.

(3) Lower Silurian Tal Formation (S_1t^1): Predominantly found in the mining area's northern region, the main lithological characteristics of this stratum are lightly metamorphosed siltstone and carbonaceous siltstone. Carbonaceous slate is observable in specific locales. Weathering usually imparts a brown-gray or light yellow-gray hue to the rocks of this layer. Without any ore content, this stratum forms the overlying layer to the Upper Yangchanggou Formation's (S_1y^2) ore-bearing system.

(4) Upper Segment of the Tal Formation (S_1t^2), Lower Silurian System: Conformably overlaying the Lower Segment of the Tal Formation, this stratum can be found within the study area's northern region. It exhibits an interlaced lithology primarily composed of carbonaceous sericite slate and carbonaceous siliceous limestone. Localized uranium mineralization occurs within this stratum, predominantly evident in gray-black carbonaceous siliceous rocks and siliceous limestone.

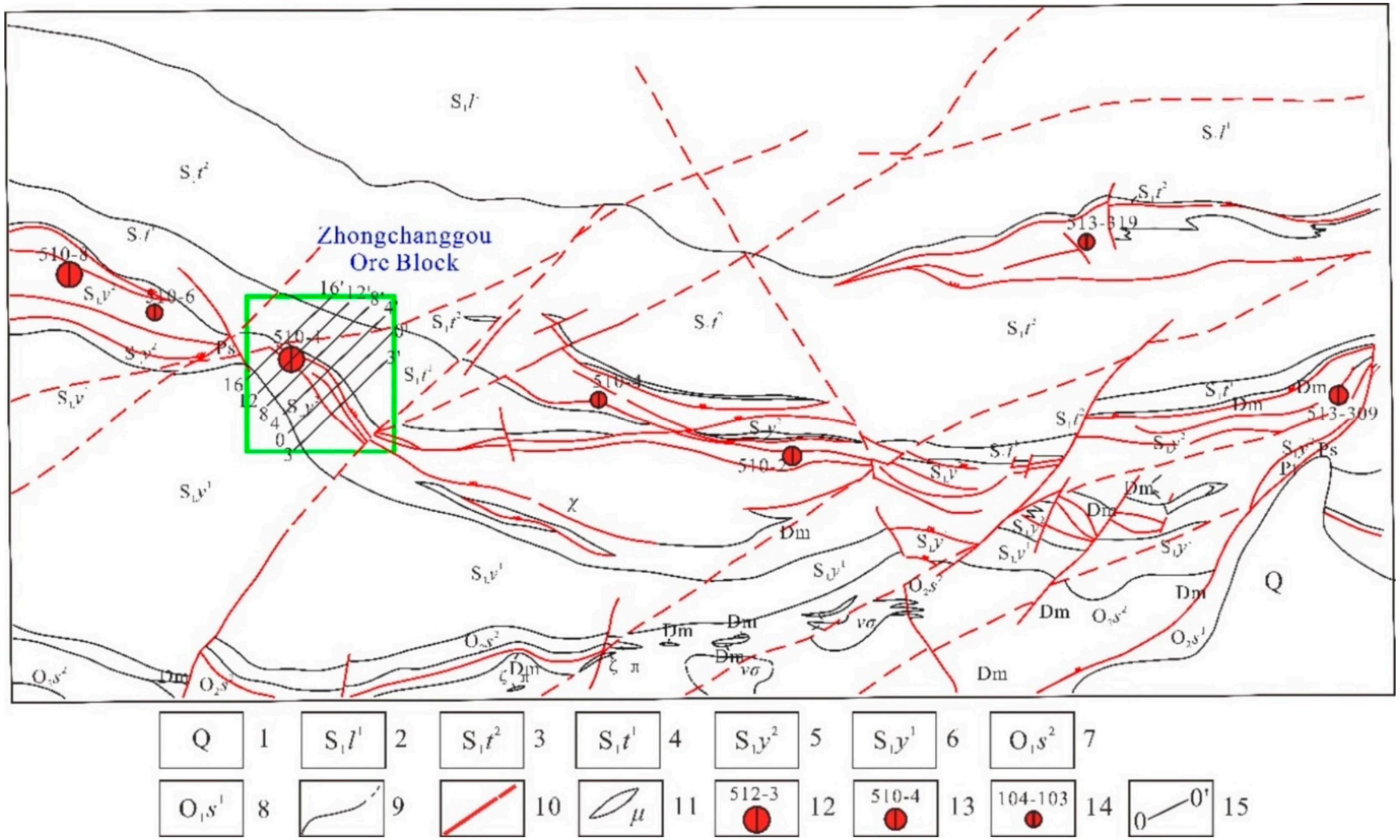

Figures S1 Schematic geology uranium deposit site points at the Zoige. 1. Quaternary series; 2. Lower part of Lalu Group; 3. Upper part of Ta'er Group; 4. Lower part of Ta'er Group; 5. Upper part of Yangchanggou Group; 6. Lower part of Yangchanggou Group; 7. Upper part of Surimutang Group; 8. Lower part of Surimutang Group; 9. Stratigraphic boundary; 10. Measured and inferred faults; 11. Uranium ore bodies; 12. Medium-sized uranium deposits; 13. Small-sized uranium deposits; 14. Uranium mineralization points; 15. Location and numbering of exploration lines.

Supplementary sediments, soils and rock geochemical characteristics

Table S1 Element content(mg/kg) and Mineral content(%)

Soil	sample	As	Cd	Cr	Cu	Ni	Pb	U	Zn	Feldspar	Muscovite	Quartz	Clay minerals	Calcite	Bernalite
XW (I)	T01	18.4	2.1	69.0	48.0	43.8	21.4	8.0	142						
	T02	21.0	1.2	72.0	38.7	39.8	23.1	5.8	129						
	T03	15.8	0.5	58.0	22.8	28.6	22.1	3.2	86						
	T04	19.2	0.4	64.0	25.0	33.3	23.5	3.0	86						
	T05	16.2	0.3	60.0	25.1	32.5	22.9	3.0	85						
	T06	18.4	1.7	62.0	32.4	35.1	25.1	3.1	112						
	T07	25.1	3.3	76.0	78.7	61.8	26.3	5.1	133						
	T08	15.6	0.5	57.0	23.5	28.5	21.4	2.9	89						
XE (II)	T26	18.7	1.9	59.0	31.7	71.5	21.9	4.8	198						
	T27	31.4	2.5	57.0	52.1	73.4	19.1	6.5	286						
	T28	14.8	0.9	62.0	29.8	34.8	22.0	5.2	118						
	T29	17.4	0.8	66.0	29.9	33.6	21.6	4.9	109						
	T30	46.7	1.1	78.0	48.7	33.3	24.1	8.7	110						
ZC (III)	T09	18.2	0.7	57.0	30.1	37.5	20.4	7.1	137		38.5	55.3	6.2		
	T10	23.8	0.7	73.0	33.6	43.0	26.7	6.5	129	17.8	22.6	52.7	6.8		
	T11	27.7	1.0	70.0	42.0	45.4	24.8	7.2	144			71	29		
	T12	23.5	0.9	68.0	33.1	37.1	23.8	20.4	172		32.2	62.8	5.1		
	T13	20.9	1.4	58.0	35.8	40.3	20.1	5.9	136						
	T14	49.9	5.8	93.0	107.5	123.5	34.5	65.1	1130		30.5	64.4		5.1	
	T15	37.7	2.5	74.0	69.3	74.7	26.1	56.4	702	21.1	17.6	55.3	6.0		

	T18	17.8	0.9	67.0	31.1	38.2	21.8	7.2	134		54.4	40.0		3.2	2.4
	T19	15.2	0.6	58.0	23.3	29.4	21.3	4.2	102						
	T20	15.8	0.5	62.0	22.4	29.3	23.1	3.4	100						
YC	T21	18.6	1.2	65.0	32.2	38.1	22.4	5.8	127						
(IV)	T22	14.2	0.6	62.0	26.9	32.2	21.2	4.6	111						
	T23	34.2	2.1	72.0	91.1	68.4	27.2	28.0	301	31.5	29.0	34.4	1.4	3.7	
	T24	26.4	0.7	67.0	38.7	36.9	21.9	4.9	105	28.2	16.8	47.3		5.1	
	T25	121.5	2.9	115.0	147.0	122.0	29.0	89.7	357	17.6	14.7	56.2	11.5		
QL	T16	9.2	1.8	47.0	31.9	53.5	14.7	23.7	215	16.4		46.3		37.3	
(V)	T17	14.4	1.5	65.0	43.7	83.9	20.9	10.4	254		54.2	40.0			5.8

Table S2 Element content(mg/kg) and Mmineral content(%)

Sediment	sample	As	Cd	Cr	Cu	Pb	Ni	U	Zn	Feldspar	Muscovite	Quartz	Clay minerals	Calcite	Sphalerite
	SX01	17.0	2.66	76	52.9	22.7	144.0	10.6	462		25.1	64.5	10.3		
	SX02	15.8	6.66	56	47.9	17.4	45.1	5.1	184		29.9	59.2		10.9	
XW (I)	SX03	21.8	6.77	54	58.0	17.3	101.0	9.4	286		32.5	59.2	2.6	15.8	
	SX04	36.8	9.33	34	36.1	10.4	142.5	93.5	441		13.6	36.5	10.3	47.4	2.4
	SX05	35.2	5.93	62	56.8	19.8	101.5	14.2	292	19.4		56.0	1.3	4.5	
XE	SX25	14.2	0.70	65	27.3	20.7	39.5	4.4	103						
(II)	SX26	69.9	3.23	56	37.7	16.2	322	20.9	441						
	SX06	17.0	1.66	51	33.8	22.9	76.0	10.0	271		38.8	54.9		6.3	
ZC	SX07	27.6	2.52	57	45.4	23.9	90.9	14.8	492			49.2	22.6	4.6	
(III)	SX08	32.9	3.59	64	57.8	23.1	117.5	21.5	785						
	SX09	69.5	9.09	76	109.5	26.7	342	55.0	2330		24.6	66.5		5.9	3.0
YC	SX12	12.6	0.63	57	27.4	21.5	36.3	4.1	104						

(IV)	SX13	15.8	1.10	58	33.3	22.5	56.8	6.0	119	28.0	21.3	38.1	12.4	0.2
	SX14	15.2	1.92	57	33.4	20.2	83.1	6.7	177	24.2	28.8	37.9	8.4	0.8
	SX15	22.0	1.68	55	35.4	20.8	154.0	12.7	657	32.7	29.1	37.6		0.6
	SX10	25.5	3.00	57	39.3	18.0	148.0	66.3	522					
QL (V)	SX11	22.1	6.52	54	43.5	17.0	176.0	17.8	1560					
	SX29	62.1	1.36	51	31.3	16.6	56.5	3.9	200					
	SX18	52.4	0.81	57	26.5	19.2	48.1	3.1	130	24.4	38.4	35.8		1.4
	SX17	14.2	4.51	55	34.7	17.2	59.7	7.2	174					
	SX19	41.3	6.73	63	234	18.3	157.5	15.5	940					
	SX20	29.5	5.09	57	48.9	19.7	171.0	11.3	314					
	SX27	33.5	2.41	53	37.1	16.4	198.0	13.9	278					
SL (VI)	SX28	38.7	4.65	59	70.4	18.9	203	12.5	496					
	SX21	32.7	6.27	56	74.2	18.0	173.0	8.8	490					
	SX22	33.4	5.46	55	53.7	18.2	107.0	10.1	703					
	SX16	19.0	1.64	52	38.4	19.2	236	11.1	991	28.3	29.2	40.2		2.2
	SX23	41.6	18.95	53	63.5	18.0	269	14.0	2100	24.9	30.1	41.2		3.9
	SX24	80.2	18.60	54	56.4	17.4	254	12.0	1925	23.6	32.1	40.5		3.7
	SX30	115.5	14.15	53	151.0	17.4	199.0	10.0	1420	41.9		51.9		6.2

Table S3 Bedrock elemental content(mg/kg)

Number	As	Cd	Cr	Cu	Ni	Pb	Zn	U
K2	20.7	11.5	27.0	218.2	81.4	6.6	479.0	612.0
K3	198.0	40.2	27.0	34.8	91.5	51.5	6015.2	23083.0
WK01	49.8	1.2	167.0	33.5	76.7	11.9	612.8	184.8
WY01	121.4	5.8	91.0	44.9	51.1	7.4	213.1	113.6

C1	165.0	23.7	133.0	243.8	302.7	8.6	1501.1	47.2
YS02	85.7	20.0	37.0	409.0	141.5	12.4	900.0	107.5
YS01	1.2	0.34	46	22.4	29.6	6.9	76	2.5

Table S4 Elemental content in sediments and soils by BCR sequential extraction(mg/kg)

Soil	sample	As				Cd				Cr				Cu			
		F4	F3	F2	F1	F4	F3	F2	F1	F4	F3	F2	F1	F4	F3	F2	F1
	T01	76.06	13.18	9.45	1.31	32.49	7.27	41.05	19.18	89.23	9.74	0.45	0.57	75.02	22.23	1.89	0.86
	T02	89.78	5.23	4.70	0.29	38.34	11.32	35.99	14.34	88.87	10.00	0.53	0.61	88.39	10.31	0.94	0.37
	T03	91.30	4.74	3.43	0.54	37.15	18.27	35.63	8.96	88.31	10.54	0.49	0.66	90.84	8.78	0.20	0.19
	T04	90.86	4.41	4.47	0.26	32.73	22.76	37.35	7.16	90.03	9.00	0.45	0.52	89.55	10.08	0.24	0.14
XW (I)	T05	89.65	5.27	4.59	0.49	25.18	24.26	41.04	9.52	88.27	10.64	0.40	0.69	89.04	10.48	0.32	0.16
	T06	88.13	5.96	5.19	0.72	42.90	10.32	38.27	8.51	91.19	7.49	0.53	0.78	89.61	9.81	0.26	0.31
	T07	92.27	3.80	3.38	0.55	46.67	9.76	33.35	10.23	91.58	7.46	0.43	0.53	82.15	16.18	1.22	0.46
	T08	90.12	4.93	4.60	0.35	28.86	16.48	42.02	12.64	91.53	7.58	0.32	0.56	88.73	10.66	0.41	0.20
	T26	86.41	8.52	4.31	0.76	40.03	8.51	39.78	11.68	89.77	9.30	0.34	0.59	79.40	19.54	0.57	0.48
	T27	88.75	6.10	3.66	1.48	38.82	8.68	29.18	23.32	87.86	11.20	0.41	0.53	71.44	24.87	2.38	1.32
XE (II)	T28	83.79	9.99	5.44	0.78	41.58	14.83	35.82	7.76	93.25	6.03	0.19	0.52	82.92	16.57	0.26	0.25
	T29	85.28	6.64	5.91	2.17	39.70	14.09	32.35	13.86	94.33	4.99	0.23	0.45	84.74	14.49	0.33	0.44
	T30	87.30	8.37	3.40	0.93	48.37	10.88	32.36	8.40	94.81	4.72	0.20	0.47	89.49	10.10	0.23	0.19
	T09	91.59	2.98	4.66	0.77	36.16	18.26	23.58	21.99	91.26	7.82	0.34	0.58	92.59	5.43	1.53	0.44
ZC (III)	T10	93.75	2.39	3.47	0.39	41.66	17.46	30.55	10.32	90.54	8.17	0.49	0.80	93.12	6.24	0.47	0.17
	T11	93.04	2.65	4.08	0.23	48.58	14.52	27.71	9.19	92.52	6.65	0.29	0.54	95.66	4.08	0.16	0.10
	T12	93.12	3.16	3.16	0.55	42.11	12.42	36.20	9.27	90.98	7.91	0.48	0.63	90.30	9.17	0.33	0.20

	T13	92.74	2.76	3.73	0.77	39.64	11.32	26.51	22.53	86.74	11.92	0.31	1.03	90.40	7.04	2.09	0.48
	T14	93.29	2.92	2.86	0.93	37.47	12.22	20.47	29.84	87.63	11.32	0.43	0.62	69.33	25.04	3.65	1.98
	T15	86.79	5.28	5.68	2.25	37.06	10.36	26.89	25.69	90.10	8.99	0.33	0.58	73.67	21.62	3.45	1.26
	T18	87.68	6.13	5.42	0.76	43.00	15.83	28.53	12.64	89.72	9.39	0.32	0.57	89.68	9.41	0.56	0.35
	T19	85.83	6.41	5.65	2.12	34.46	15.00	36.60	13.94	89.40	9.65	0.32	0.63	88.25	10.87	0.46	0.42
	T20	92.44	3.39	3.45	0.71	30.99	21.88	35.67	11.47	90.17	8.58	0.60	0.65	89.38	9.94	0.37	0.31
YC	T21	91.21	4.12	4.17	0.50	40.78	11.28	33.16	14.78	91.82	7.10	0.51	0.57	88.92	10.17	0.54	0.37
(IV)	T22	86.19	7.08	5.20	1.53	32.95	17.95	38.20	10.91	95.00	4.13	0.46	0.41	88.73	10.56	0.36	0.34
	T23	92.33	3.13	3.58	0.97	47.14	13.60	26.73	12.54	84.49	14.47	0.37	0.66	80.18	18.28	1.10	0.45
	T24	88.77	6.85	3.47	0.91	29.34	48.55	14.96	7.14	87.79	11.01	0.50	0.71	62.49	34.96	2.01	0.55
	T25	93.24	4.23	2.18	0.35	21.24	20.09	23.90	34.77	86.26	12.84	0.28	0.62	81.12	9.92	5.31	3.65
QL	T16	65.08	11.39	21.35	2.18	43.81	17.07	26.15	12.96	89.27	9.72	0.35	0.66	69.68	29.72	0.10	0.50
(V)	T17	88.07	5.78	5.15	1.00	36.38	8.44	32.79	22.39	88.85	10.20	0.32	0.63	72.00	25.57	1.23	1.19
	SX01	0.26	0.09	0.14	0.51	0.55	0.05	0.38	0.01	95.07	4.27	0.28	0.39	84.36	13.71	0.64	1.29
	SX02	0.23	0.12	0.11	0.54	0.69	0.09	0.21	0.01	93.78	5.20	0.29	0.73	84.42	14.14	0.59	0.84
XW<br>(I)	SX03	0.23	0.14	0.28	0.36	0.49	0.17	0.34	0.00	93.86	5.15	0.31	0.68	82.80	14.91	0.93	1.35
	SX04	0.06	0.27	0.36	0.31	0.34	0.22	0.43	0.01	90.53	8.06	0.28	1.12	81.07	18.71	0.04	0.18
	SX05	0.08	0.10	0.51	0.31	0.34	0.34	0.32	0.00	94.38	4.80	0.28	0.54	85.84	12.69	0.78	0.70
XE	SX25	0.19	0.12	0.41	0.28	0.49	0.08	0.42	0.01	95.37	4.16	0.12	0.35	92.77	5.37	1.14	0.72
(II)	SX26	0.28	0.06	0.31	0.35	0.58	0.06	0.34	0.01	93.62	5.66	0.17	0.55	83.91	15.58	0.05	0.45
	SX06	0.06	0.05	0.64	0.26	0.37	0.14	0.49	0.00	94.42	4.65	0.26	0.67	92.55	6.09	0.59	0.78
ZC	SX07	0.10	0.33	0.41	0.16	0.30	0.16	0.53	0.00	94.67	4.51	0.24	0.58	88.74	9.78	0.72	0.76
(III)	SX08	0.09	0.09	0.44	0.38	0.42	0.16	0.41	0.01	95.23	3.96	0.27	0.53	88.20	9.50	1.13	1.16
	SX09	0.12	0.16	0.30	0.41	0.71	0.12	0.17	0.00	96.78	2.59	0.18	0.46	93.95	4.79	0.42	0.84
YC	SX12	0.13	0.05	0.66	0.15	0.41	0.10	0.49	0.00	94.78	4.47	0.28	0.46	93.91	4.80	0.68	0.60

(IV)	SX13	0.17	0.12	0.47	0.24	0.53	0.11	0.36	0.00	94.94	4.30	0.32	0.44	93.47	4.01	1.31	1.22
	SX14	0.12	0.44	0.24	0.20	0.48	0.11	0.40	0.01	95.40	3.89	0.27	0.44	93.05	4.77	1.22	0.96
	SX15	0.19	0.06	0.42	0.33	0.51	0.06	0.42	0.01	94.89	4.38	0.25	0.48	91.15	6.09	1.59	1.17
	SX10	0.08	0.10	0.49	0.32	0.67	0.16	0.17	0.00	93.22	6.03	0.11	0.64	81.04	18.50	0.04	0.43
QL (V)	SX11	0.07	0.31	0.36	0.26	0.77	0.15	0.08	0.00	92.07	7.11	0.13	0.69	78.46	21.13	0.11	0.30
	SX29	0.19	0.09	0.46	0.26	0.51	0.09	0.39	0.01	94.33	4.99	0.23	0.45	90.01	7.70	1.13	1.15
	SX18	0.19	0.06	0.46	0.29	0.50	0.08	0.42	0.00	94.39	4.91	0.26	0.44	90.51	7.74	1.01	0.75
	SX17	0.37	0.11	0.23	0.29	0.51	0.13	0.35	0.00	93.81	5.44	0.16	0.60	87.13	12.36	0.06	0.46
	SX19	0.16	0.13	0.28	0.43	0.44	0.19	0.37	0.01	94.34	4.88	0.32	0.47	78.81	9.51	4.42	7.25
	SX20	0.11	0.11	0.43	0.34	0.37	0.13	0.50	0.00	93.98	5.33	0.13	0.56	87.01	12.34	0.14	0.51
	SX27	0.16	0.12	0.30	0.42	0.50	0.06	0.43	0.01	93.49	5.62	0.27	0.61	84.57	14.63	0.23	0.58
SL (VI)	SX28	0.30	0.12	0.20	0.38	0.63	0.12	0.24	0.01	93.25	6.03	0.19	0.52	80.69	18.30	0.35	0.66
	SX21	0.10	0.31	0.43	0.16	0.43	0.16	0.41	0.00	94.01	5.19	0.26	0.53	82.83	14.06	1.37	1.73
	SX22	0.15	0.16	0.47	0.22	0.61	0.12	0.26	0.00	94.30	4.98	0.24	0.47	84.68	12.78	1.59	0.94
	SX16	0.15	0.24	0.29	0.32	0.45	0.11	0.43	0.01	94.77	4.43	0.26	0.55	89.91	6.23	2.30	1.56
	SX23	0.12	0.05	0.53	0.30	0.63	0.09	0.28	0.00	94.22	5.04	0.21	0.53	91.43	2.46	4.28	1.83
	SX24	0.12	0.13	0.46	0.29	0.48	0.09	0.42	0.00	85.29	13.95	0.25	0.51	56.65	38.55	2.40	2.40
	SX30	0.16	0.04	0.25	0.56	0.47	0.09	0.43	0.01	94.81	4.72	0.20	0.47	77.54	14.91	3.51	4.04

Table S5 Elemental content in sediments and soils by BCR sequential extraction(mg/kg)

Soil	sample	Ni				Pb				U				Zn			
		F4	F3	F2	F1	F4	F3	F2	F1	F4	F3	F2	F1	F4	F3	F2	F1
XW	T01	71.91	12.20	10.23	5.67	51.18	18.82	29.73	0.28	92.98	6.32	0.38	0.32	83.45	5.13	6.03	5.38

(I)	T02	86.06	7.26	4.55	2.13	62.65	15.94	21.24	0.17	94.51	4.91	0.30	0.28	94.22	2.71	1.90	1.17
	T03	87.97	7.84	3.12	1.07	74.46	18.89	6.50	0.14	92.51	6.56	0.47	0.46	94.30	2.07	2.43	1.19
	T04	85.90	8.99	4.11	1.01	74.73	19.53	5.66	0.08	92.31	6.74	0.48	0.47	94.39	3.09	1.87	0.65
	T05	85.15	8.91	4.54	1.39	71.67	20.65	7.51	0.17	91.91	7.08	0.51	0.50	93.80	2.46	2.57	1.17
	T06	84.05	10.55	4.04	1.36	73.87	20.16	5.85	0.11	88.22	10.30	0.74	0.74	93.19	2.80	3.05	0.96
	T07	84.74	8.58	5.14	1.54	78.08	15.25	6.58	0.09	88.31	10.34	0.68	0.66	94.28	2.85	2.09	0.77
	T08	85.06	8.15	4.98	1.81	58.99	18.85	22.00	0.15	91.09	7.80	0.56	0.55	92.54	3.15	2.69	1.62
	T26	58.19	21.39	15.78	4.63	53.94	22.67	23.28	0.11	92.25	6.92	0.42	0.41	84.30	5.90	6.27	3.54
	T27	62.76	17.73	12.89	6.62	57.02	15.56	27.21	0.21	93.61	5.71	0.36	0.32	74.34	10.34	8.18	7.14
XE	T28	74.95	18.35	5.24	1.46	65.15	27.27	7.44	0.14	93.39	5.97	0.33	0.32	89.42	5.30	3.65	1.62
(II)	T29	81.45	11.08	5.10	2.37	57.58	15.53	26.64	0.25	92.37	6.78	0.44	0.42	86.78	5.07	4.82	3.33
	T30	87.51	8.01	3.32	1.16	91.31	4.47	4.13	0.09	94.11	5.34	0.28	0.27	93.81	3.15	2.11	0.93
	T09	83.83	6.52	5.99	3.67	66.11	9.47	24.27	0.16	93.71	5.56	0.37	0.37	86.53	4.88	5.72	2.87
	T10	90.15	5.78	2.94	1.13	83.50	11.40	5.03	0.07	93.84	5.52	0.33	0.31	93.40	2.47	2.56	1.57
ZC	T11	90.27	5.89	2.78	1.06	84.44	9.36	6.09	0.10	95.14	4.33	0.27	0.26	95.59	1.73	1.65	1.03
(III)	T12	84.04	9.27	5.04	1.66	80.99	13.10	5.81	0.10	96.11	3.65	0.13	0.11	85.37	4.34	6.41	3.88
	T13	84.45	6.05	6.77	2.74	64.83	12.30	22.75	0.13	90.67	8.37	0.48	0.48	86.45	5.17	5.38	3.01
	T14	69.52	17.49	7.11	5.88	80.22	5.16	14.44	0.18	86.07	12.52	0.61	0.81	64.24	12.97	10.86	11.93
	T15	67.54	13.79	11.74	6.93	75.03	6.04	18.79	0.13	90.42	8.74	0.60	0.24	56.29	10.34	17.09	16.28
	T18	86.35	8.15	3.72	1.79	73.16	17.26	9.35	0.22	90.25	8.70	0.54	0.51	92.71	3.18	2.29	1.81
	T19	85.48	7.67	4.75	2.10	59.08	15.79	24.88	0.25	93.50	5.73	0.39	0.38	91.12	3.01	3.56	2.31
YC	T20	86.88	8.09	3.56	1.47	71.95	21.34	6.54	0.17	93.14	6.02	0.42	0.42	93.83	3.60	1.65	0.93
(IV)	T21	85.01	7.65	4.95	2.39	59.95	15.18	24.70	0.18	93.51	5.76	0.37	0.36	91.00	3.54	2.97	2.49
	T22	82.88	10.26	5.07	1.79	60.07	18.41	21.34	0.17	93.07	6.14	0.40	0.39	91.93	2.79	3.31	1.97
	T23	78.68	10.98	7.82	2.52	66.86	14.21	18.85	0.08	72.84	25.70	0.83	0.63	73.21	19.40	5.31	2.08

	T24	85.06	8.00	4.82	2.12	71.06	5.33	23.48	0.13	85.06	14.02	0.47	0.45	47.46	51.04	0.91	0.60
	T25	23.26	35.90	13.80	27.05	94.42	4.31	1.22	0.06	98.84	0.66	0.23	0.27	43.91	37.26	4.15	14.68
QL	T16	62.77	28.25	3.22	5.76	72.26	25.90	1.69	0.16	94.88	4.75	0.09	0.28	78.00	11.69	6.45	3.86
(V)	T17	58.93	16.21	15.53	9.33	46.52	18.90	34.24	0.34	94.82	4.69	0.28	0.21	77.05	7.41	7.37	8.16
	SX01	73.82	9.29	9.05	7.85	63.93	11.09	24.59	0.39	94.71	4.71	0.30	0.28	82.44	3.66	6.52	7.39
XW	SX02	78.66	7.57	6.15	7.61	78.79	12.69	7.76	0.77	91.96	7.13	0.43	0.48	87.29	2.69	4.86	5.16
(I)	SX03	73.61	8.45	9.95	8.00	81.90	9.33	8.39	0.38	93.49	5.82	0.28	0.41	84.21	3.26	6.60	5.93
	SX04	77.78	16.49	1.03	4.71	92.70	6.95	0.25	0.10	93.74	5.28	0.09	0.89	86.73	8.51	2.12	2.63
	SX05	77.92	6.64	8.35	7.09	82.33	9.23	8.15	0.29	94.86	4.70	0.19	0.25	86.34	2.87	5.68	5.12
XE	SX25	89.35	3.09	3.64	3.92	66.10	8.85	24.55	0.50	90.05	8.79	0.59	0.57	94.71	1.22	1.55	2.52
(II)	SX26	78.39	11.41	3.99	6.21	90.81	7.81	1.25	0.13	95.23	4.41	0.07	0.30	89.43	4.39	2.90	3.28
	SX06	82.37	5.53	5.80	6.30	67.14	10.80	21.45	0.61	94.41	4.94	0.26	0.39	87.36	2.53	5.01	5.10
ZC	SX07	81.41	6.71	5.95	5.94	71.47	10.24	17.97	0.32	94.97	4.58	0.19	0.27	84.84	3.67	5.91	5.58
(III)	SX08	78.06	6.72	6.65	8.58	88.62	5.14	6.10	0.14	96.12	3.49	0.15	0.24	82.53	3.86	5.66	7.95
	SX09	88.07	2.75	4.69	4.49	93.08	4.70	2.16	0.06	98.05	1.72	0.05	0.18	90.89	1.76	2.82	4.53
	SX12	90.13	2.85	3.19	3.83	62.97	9.54	26.99	0.49	90.86	8.05	0.54	0.54	95.57	1.17	1.22	2.03
YC	SX13	83.88	3.25	4.73	8.14	62.39	6.90	30.02	0.69	89.61	9.10	0.65	0.64	93.95	1.07	1.48	3.50
(IV)	SX14	80.45	3.62	7.53	8.40	73.15	2.02	24.54	0.29	93.75	5.49	0.41	0.35	88.27	1.71	3.24	6.79
	SX15	70.46	4.09	9.01	16.44	73.85	3.01	22.61	0.53	94.05	5.23	0.41	0.31	75.48	1.36	5.09	18.07
	SX10	79.04	13.31	2.20	5.44	96.21	2.93	0.65	0.21	94.14	5.27	0.03	0.56	87.69	5.44	3.01	3.86
QL	SX11	72.55	12.76	5.70	8.99	87.60	10.62	1.67	0.12	94.14	5.08	0.08	0.70	82.99	4.62	5.31	7.07
(V)	SX29	84.77	4.40	4.50	6.34	82.80	8.92	7.93	0.35	91.94	6.97	0.51	0.58	90.39	2.37	2.74	4.51
	SX18	83.21	4.30	6.70	5.80	84.98	8.26	6.46	0.29	88.40	10.11	0.75	0.73	93.55	1.97	2.13	2.35
SL	SX17	81.90	10.18	2.65	5.27	81.69	16.49	1.59	0.24	93.98	5.47	0.20	0.35	92.93	2.95	1.90	2.23
(VI)	SX19	86.11	8.93	1.82	3.13	88.34	5.30	5.71	0.65	95.16	4.20	0.36	0.29	88.90	2.70	1.57	6.83

SX20	74.39	9.81	7.36	8.44	86.65	9.60	3.52	0.23	94.42	5.16	0.14	0.27	89.02	3.50	3.81	3.67
SX27	76.18	9.31	6.84	7.68	84.76	10.45	4.58	0.20	94.71	4.66	0.13	0.50	90.52	3.17	3.12	3.18
SX28	76.30	10.20	7.19	6.31	81.13	12.67	5.92	0.27	94.72	4.77	0.14	0.37	88.69	4.18	4.11	3.01
SX21	73.52	6.92	7.86	11.70	84.47	6.83	8.12	0.58	94.45	4.90	0.27	0.39	85.09	2.87	4.44	7.60
SX22	78.95	7.59	6.60	6.86	88.93	3.97	6.76	0.34	94.50	4.92	0.30	0.29	83.31	2.73	5.33	8.63
SX16	68.13	3.15	10.56	18.16	72.50	3.82	23.23	0.46	94.24	4.94	0.44	0.37	75.70	1.30	5.50	17.50
SX23	71.55	0.28	18.43	9.73	80.66	9.71	9.06	0.56	97.62	1.58	0.31	0.48	75.86	0.05	12.35	11.73
SX24	77.90	2.75	8.45	10.89	94.08	0.79	4.84	0.29	52.03	46.86	0.41	0.70	81.96	0.69	5.59	11.75
SX30	75.30	9.15	7.21	8.34	88.23	5.27	6.06	0.45	94.63	4.59	0.31	0.47	81.61	3.40	4.97	10.02

Supplementary Geoaccumulation index assessment data

Table S6 Geoaccumulation index of sediments and soils

	As	Cd	Cr	Cu	Ni	Pb	Zn	U
S1	0.12	4.49	-0.64	0.18	1.56	-1.03	1.83	1.21
S2	0.02	5.81	-1.08	0.04	-0.12	-1.41	0.50	0.16
S3	0.48	5.84	-1.13	0.31	1.05	-1.42	1.14	1.04
S4	1.24	6.30	-1.80	-0.37	1.54	-2.16	1.77	4.35
S5	1.17	5.65	-0.93	0.28	1.05	-1.23	1.17	1.63
S6	0.12	3.81	-1.22	-0.46	0.64	-1.02	1.06	1.13
S7	0.82	4.41	-1.06	-0.04	0.89	-0.96	1.92	1.69
S8	1.08	4.92	-0.89	0.31	1.26	-1.00	2.60	2.23
S9	2.16	6.26	-0.64	1.23	2.81	-0.80	4.17	3.59
S10	0.71	4.66	-1.06	-0.25	1.60	-1.36	2.01	3.86
S11	0.50	5.78	-1.13	-0.10	1.85	-1.45	3.59	1.96
S12	-0.31	2.41	-1.06	-0.77	-0.43	-1.11	-0.32	-0.16
S13	0.02	3.21	-1.03	-0.49	0.22	-1.04	-0.12	0.39
S14	-0.04	4.02	-1.06	-0.48	0.77	-1.20	0.45	0.55
S15	0.50	3.83	-1.11	-0.40	1.66	-1.16	2.34	1.47
S16	0.28	3.79	-1.19	-0.28	2.27	-1.27	2.93	1.28
S17	-0.14	5.25	-1.11	-0.43	0.29	-1.43	0.42	0.65
S18	1.75	2.77	-1.06	-0.82	-0.02	-1.27	0.00	-0.56
S19	1.40	5.83	-0.91	2.33	1.69	-1.34	2.86	1.76
S20	0.92	5.42	-1.06	0.07	1.81	-1.23	1.28	1.30

S21	1.07	5.73	-1.08	0.67	1.82	-1.36	1.92	0.94
S22	1.10	5.53	-1.11	0.20	1.13	-1.35	2.44	1.14
S23	1.42	7.32	-1.16	0.44	2.46	-1.36	4.02	1.61
S24	2.36	7.29	-1.13	0.27	2.38	-1.41	3.89	1.39
S25	-0.14	2.56	-0.87	-0.77	-0.31	-1.16	-0.33	-0.06
S26	2.16	4.77	-1.08	-0.31	2.72	-1.52	1.77	2.19
S27	1.10	4.35	-1.16	-0.33	2.02	-1.50	1.10	1.60
S28	1.31	5.29	-1.01	0.59	2.05	-1.29	1.93	1.45
S29	1.99	3.52	-1.22	-0.58	0.21	-1.48	0.62	-0.23
S30	2.89	6.90	-1.16	1.69	2.02	-1.41	3.45	1.13
T1	0.24	4.14	-0.78	0.04	-0.16	-1.11	0.13	0.81
T2	0.43	3.35	-0.72	-0.27	-0.30	-1.00	-0.01	0.34
T3	0.02	2.05	-1.03	-1.03	-0.77	-1.07	-0.59	-0.52
T4	0.30	1.68	-0.89	-0.90	-0.55	-0.98	-0.59	-0.61
T5	0.05	1.52	-0.98	-0.89	-0.59	-1.02	-0.61	-0.61
T6	0.24	3.88	-0.93	-0.53	-0.48	-0.88	-0.21	-0.56
T7	0.69	4.80	-0.64	0.75	0.34	-0.82	0.04	0.16
T8	0.00	2.19	-1.06	-0.99	-0.78	-1.11	-0.54	-0.66
T9	0.22	2.60	-1.06	-0.63	-0.38	-1.18	0.08	0.63
T10	0.61	2.58	-0.70	-0.47	-0.19	-0.80	-0.01	0.51
T11	0.83	3.13	-0.76	-0.15	-0.11	-0.90	0.15	0.65
T12	0.59	2.97	-0.80	-0.50	-0.40	-0.96	0.41	2.16
T13	0.42	3.60	-1.03	-0.38	-0.28	-1.21	0.07	0.37
T14	1.68	5.62	-0.35	1.20	1.34	-0.43	3.12	3.83
T15	1.27	4.40	-0.68	0.57	0.61	-0.83	2.44	3.62

T16	-0.76	3.89	-1.33	-0.55	0.13	-1.66	0.73	2.37
T17	-0.12	3.63	-0.87	-0.09	0.78	-1.15	0.97	1.18
T18	0.19	2.93	-0.82	-0.58	-0.36	-1.09	0.05	0.65
T19	-0.04	2.41	-1.03	-1.00	-0.73	-1.12	-0.35	-0.12
T20	0.02	2.02	-0.93	-1.06	-0.74	-1.00	-0.38	-0.43
T21	0.25	3.35	-0.87	-0.53	-0.36	-1.05	-0.03	0.34
T22	-0.14	2.24	-0.93	-0.79	-0.60	-1.13	-0.23	0.01
T23	1.13	4.15	-0.72	0.97	0.48	-0.77	1.21	2.61
T24	0.76	2.60	-0.82	-0.27	-0.41	-1.08	-0.31	0.10
T25	2.96	4.61	-0.04	1.66	1.32	-0.68	1.46	4.29
T26	0.26	3.99	-1.01	-0.56	0.55	-1.08	0.61	0.07
T27	1.01	4.40	-1.06	0.16	0.59	-1.28	1.14	0.51
T28	-0.08	2.93	-0.93	-0.65	-0.49	-1.08	-0.14	0.18
T29	0.16	2.70	-0.84	-0.64	-0.54	-1.10	-0.25	0.10
T30	1.58	3.27	-0.60	0.06	-0.55	-0.94	-0.24	0.93

Table S7 Statistical analysis of geoaccumulation index evaluation results for surface soils

Metals	Sample	As	Cd	Cr	Cu	Ni	Pb	Zn	U
I-Geo	Ave.	0.49	3.25	-0.84	-0.27	-0.12	-1.02	0.27	0.76
	Max.	2.96	5.62	-0.04	1.66	1.34	-0.43	3.12	4.29
	Min.	-0.76	1.52	-1.33	-1.06	-0.78	-1.66	-0.61	-0.66
	Med.	0.26	3.20	-0.87	-0.51	-0.36	-1.06	-0.01	0.35
Proportion of pollution levels	non-polluted	20%	—	100%	73.33%	70%	100%	50%	23.34%

	lightly to moderately	60%	—	—	20%	23.33%	—	33.34%	53.33%
	moderate	16.67%	6.67%	—	6.67%	6.67%	—	10%	3.33%
	moderate-strong	3.33%	40%	—	—	—	—	3.33%	10%
	strong	—	30%	—	—	—	—	3.33%	6.67%
	strong-extremely	—	20%	—	—	—	—	—	3.33%
	extremely	—	3.33%	—	—	—	—	—	—

Table S8 Statistical analysis of geoaccumulation index evaluation results for Sediments

Metals	Sample	As	Cd	Cr	Cu	Ni	Pb	Zn	U
I-Geo	Ave.	0.94	4.92	-1.07	0.06	1.30	-1.29	1.75	1.36
	Max.	2.89	7.32	-0.64	2.33	2.81	0.80	4.17	4.35
	Min.	-0.31	2.41	-1.80	-0.82	-0.43	-2.16	-0.33	-0.56
	Med.	0.99	5.09	-1.08	-0.07	1.55	-1.32	1.80	1.29
Proportion of pollution levels	non-polluted	13.33%	—	100%	53.33%	13.33%	100%	13.33%	13.33%
	lightly to moderately	36.67%	—	—	36.67%	20%	—	13.33%	16.67%
	moderate	36.67%	—	—	6.67%	40%	—	36.67%	53.33%
	moderate-strong	13.33%	10%	—	3.33%	26.67%	—	20%	6.67%
	strong	—	16.67%	—	—	—	—	10%	6.67%
	strong-extremely	—	23.33%	—	—	—	—	6.67%	3.33%
	extremely	—	50%	—	—	—	—	—	—

Supplementary Pollution Load Index assessment

Table S9 Evaluation results of pollution load index in surface soils of the study area.

Sample	PLI	Sample	PLI	Sample	PLI
T01	2.00	T11	1.92	T21	1.65
T02	1.76	T12	2.03	T22	1.31
T03	1.16	T13	1.72	T23	3.29
T04	1.20	T14	6.01	T24	1.58
T05	1.14	T15	4.03	T25	5.79
T06	1.57	T16	1.92	T26	1.92
T07	2.38	T17	2.18	T27	2.41
T08	1.16	T18	1.63	T28	1.47
T09	1.54	T19	1.26	T29	1.45
T10	1.71	T20	1.21	T30	2.03

Table S10 Statistical evaluation of pollution load index in surface soils of the study area.

	Baseline pollution	Moderate pollution	Significant pollution	Extremely high pollution	Total
Number	0	21	5	4	30
Proportion(%)	0	70	16.67	13.33	100

Table S11 Evaluation results of pollution load index in sediment of the study area.

Sample	PLI	Sample	PLI	Sample	PLI

S01	2.93	S11	3.89	S21	3.48
S02	2.11	S12	1.29	S22	3.29
S03	2.82	S13	1.66	S23	5.38
S04	3.85	S14	1.95	S24	5.52
S05	3.22	S15	2.78	S25	1.37
S06	2.13	S16	2.95	S26	3.79
S07	2.92	S17	2.03	S27	2.79
S08	3.73	S18	1.61	S28	3.67
S09	7.63	S19	4.88	S29	1.92
S10	3.62	S20	3.13	S30	5.75

Table S12 Statistical evaluation of pollution load index in sediments of the study area.

	Baseline pollution	Moderate pollution	Significant pollution	Extremely high pollution	Total
Number	0	6	9	15	30
Proportion(%)	0	20	30	50	100

Supplementary Health risk assessment model

The exposure levels for each pathway are calculated as shown in (9) ~ (11).

$$ADD_{ing}=\frac{CS\times IR\times TF\times EF\times ED}{BW\times AT} \quad (9)$$

$$ADD_{der}=\frac{CS\times AF\times TF\times SA\times ABS\times EF\times ED}{BW\times AT} \quad (10)$$

$$ADD_{inh}=\frac{CS\times IAR\times EF\times ED}{BW\times AT\times PEF} \quad (11)$$

Analyzed from the perspective of PETs, chronic non-carcinogenic health concerns include Cr, Ni, Cu, As, Pb, Zn, and Cd, while Cr, Ni, As, and Cd are carcinogenic. The detailed calculations are as follows in (12) ~ (15):

$$HQ_i=\sum\frac{ADD_i}{RfD_i} \quad (12)$$

$$HI=\sum HQ_i \quad (13)$$

$$CR_i=\sum ADD_i\times SF_i \quad (14)$$

$$TRC=\sum CR_i \quad (15)$$

In the formula, *RfD* represents the reference dose for heavy metal element *I* through exposure pathway *j,* mg(kg·d)$^{-1}$ and SF is the carcinogenic slope factor of PETs in different exposure pathways, (kg·d)·mg^{-1}.

Table S13 Exposure parameters related to health risk assessment model

Parameters	Meaning	Value		Reference
		Adults	Children	
CS	Pollutant concentration of soil (mg/kg)	—	—	This Study
IR	Soil ingestion rate(mg/d)	100	200	(Chen，2020)

AF	skin adhesion factor(kg/cm^2)	0.2	0.2	(Fang et al.，2021)
TF	Conversion factor(kg/mg)	1×10^{-6}	1×10^{-6}	
EF	Exposure frequency(d/a)	180	180	(Chen，2020)
ED	Exposure duration(a)	24	6	
BW	The average body weight(kg)	62	15.9	
AT	Average exposure time(d)	ED×365(non-carcinogenic)、70×365(carcinogenic)		
SA	Exposure of the skin surface area(cm^2)	1.6×10^4	2800	
ABS	Skin absorption factor	As：0.03、Cu：0.06、Pb：0.006、Zn：0.02、Cd：0.001、Ni：0.091、Cr：0.04		(Cheng et al., 2022)
IAR	Inhalation rate(m^3/d)	16.1	8.3	(Zhang et al., 2024)
PEF	Particle emission factor(m^3/kg)	1.36×10^9	1.36×10^9	

Note: — indicates no relevant data available in the article

Table S14 RfD and SF values of heavy metals in health risk assessment

Metals	As	Cd	Cr	Cu	Ni	Pb	Zn
RfDs	3.00E-03	1.00E-03	1.50E+00	4.00E-02	2.00E-02	3.50E-03	3.00E-01
RfDd	3.83E-06	2.50E-05	1.95E-02	1.20E-02	8.00E-04	5.20E-03	6.00E-02
RfDi	1.23E-04	1.00E-03	2.86E-05	4.02E-02	2.06E-02	5.25E-03	3.00E-01
SF_s	1.51E+01	6.30E+00	4.20E+01	—	8.40E-01	—	—
SF_d	1.50E+00	6.10E+00	5.46E-01	—	3.30E-02	—	—
SF_i	4.30E-03	1.80E-03	4.20E+01	—	8.40E-01	—	—

Note: — indicates no relevant data available in the article

Table S15 Non-carcinogenic health risk assessment results

Metals	Object	ADD_{ing}	ADD_{der}	ADD_{inh}	HQ	HI	
						Adult	Child
As	Adult	2.04E-05	1.95E-05	2.41E-09	5.11E+00	5.27E+00	3.72E+00
	Child	1.59E-04	1.33E-05	4.84E-09	3.52E+00		
Cd	Adult	1.15E-06	3.66E-08	1.36E-10	2.61E-03		
	Child	8.93E-06	2.50E-08	2.73E-10	9.93E-03		
Cr	Adult	5.34E-05	6.83E-05	6.32E-09	3.76E-03		

	Child	4.16E-04	4.66E-05	1.27E-08	3.11E-03
Cu	Adult	3.52E-05	6.75E-05	4.16E-09	6.50E-03
	Child	2.74E-04	4.61E-05	8.37E-09	1.07E-02
Ni	Adult	3.93E-05	1.15E-04	4.66 E-09	1.45E-01
	Child	3.07E-04	7.82E-05	9.36E-09	1.13E-01
Pb	Adult	1.84E-05	3.53E-06	2.18E-09	5.94E-03
	Child	1.44E-04	2.41E-06	4.38E-09	4.14E-02
Zn	Adult	1.60E-04	1.02E-04	1.90E-08	2.24E-03
	Child	1.25E-03	6.99E-05	3.81E-08	5.34E-03

Table S16 Carcinogenic health risk assessment results

Metals	Object	ADD_{ing}	ADD_{der}	ADD_{inh}	CR	TCR	
						Adult	Child
As	Adult	6.98E-06	6.70E-06	8.26E-10	1.15E-04	9.12E-04	1.73E-03
	Child	1.36E-05	1.14E-06	4.15E-10	2.07E-04		
Cd	Adult	3.93E-07	1.26E-08	4.65E-11	2.55E-06		
	Child	7.65E-07	2.14E-09	2.34E-11	4.84E-06		
Cr	Adult	1.83E-05	2.34E-06	2.17E-09	7.81E-04		
	Child	3.57E-05	4.00E-06	1.09E-09	1.50E-03		

Ni	Adult	1.35E-05	3.93E-06	1.60E-09	1.26E-05
	Child	2.63E-05	6.70E-06	8.02E-10	2.23E-05
